# Multi-omics bioactivity profile-based chemical grouping and read-across: a case study with *Daphnia magna* and azo dyes

**DOI:** 10.1007/s00204-024-03759-6

**Published:** 2024-05-02

**Authors:** Hanna Gruszczynska, Rosemary E. Barnett, Gavin R. Lloyd, Ralf J. M. Weber, Thomas N. Lawson, Jiarui Zhou, Elena Sostare, John K. Colbourne, Mark R. Viant

**Affiliations:** 1 School of Biosciences, University of Birmingham, Edgbaston, Birmingham, B15 2TT UK; 2 Michabo Health Science Limited, Union House, 111 New Union Street, Coventry, CV1 2NT UK; 3 Phenome Centre Birmingham, University of Birmingham, Edgbaston, Birmingham, B15 2TT UK

**Keywords:** NAM, Omics, Multi-omics, Bioactivity similarity, Bioactivity profile-based grouping, Replicability confidence

## Abstract

Grouping/read-across is widely used for predicting the toxicity of data-poor target substance(s) using data-rich source substance(s). While the chemical industry and the regulators recognise its benefits, registration dossiers are often rejected due to weak analogue/category justifications based largely on the structural similarity of source and target substances. Here we demonstrate how multi-omics measurements can improve confidence in grouping via a statistical assessment of the similarity of molecular effects. Six azo dyes provided a pool of potential source substances to predict long-term toxicity to aquatic invertebrates (*Daphnia magna*) for the dye Disperse Yellow 3 (DY3) as the target substance. First, we assessed the structural similarities of the dyes, generating a grouping hypothesis with DY3 and two Sudan dyes within one group. *Daphnia magna* were exposed acutely to equi-effective doses of all seven dyes (each at 3 doses and 3 time points), transcriptomics and metabolomics data were generated from 760 samples. Multi-omics bioactivity profile-based grouping uniquely revealed that Sudan 1 (S1) is the most suitable analogue for read-across to DY3. Mapping ToxPrint structural fingerprints of the dyes onto the bioactivity profile-based grouping indicated an aromatic alcohol moiety could be responsible for this bioactivity similarity. The long-term reproductive toxicity to aquatic invertebrates of DY3 was predicted from S1 (21-day NOEC, 40 µg/L). This prediction was confirmed experimentally by measuring the toxicity of DY3 in *D. magna*. While limitations of this ‘omics approach are identified, the study illustrates an effective statistical approach for building chemical groups.

**Supplementary Information:**

The online version contains supplementary material available at 10.1007/s00204-024-03759-6.

## Introduction

Grouping/read-across (G/RAx) is a widely used alternative (non-animal) method for chemical risk assessment, utilising knowledge of a well-characterised source substance to predict the toxicity of a target substance. Traditionally, the first step is to group substances into a chemical category based on similar structural parameters and ideally similar toxicological responses and/or shared metabolism, described as a grouping hypothesis. The second step is to read across the known toxicity data–for a specific endpoint–from the source to the target, often referred to as data-gap filling. G/RAx has been used to predict at least one toxicological endpoint in up to 75% of chemical registration dossiers submitted to the European Chemicals Agency (ECHA), frequently being used for ecotoxicological and higher-tier human health endpoints (European Chemicals Agency 2020, 2023). In the US Environmental Protection Agency’s (EPA) High Production Volume Chemicals Challenge Program, this alternative method has satisfied 55% of toxicity endpoints across 1,420 chemicals, which would otherwise have required extensive animal testing (Bishop et al. 2012). G/RAx is supported by extensive guidance, for example the Organisation for Economic Cooperation and Development (OECD) Guidance on Grouping of Chemicals No. 194 (OECD 2017) and ECHA’s Read-Across Assessment Framework (RAAF; European Chemicals Agency 2015). The latter describes how to construct and report an (eco)toxicological G/RAx as part of a regulatory submission under REACH legislation Annex XI.

While the chemical industry, regulators and policymakers recognise the benefits of G/RAx as reducing time, costs and use of animals compared to standardised toxicity testing guidelines, several challenges to its effective implementation remain, particularly in forming a robust grouping hypothesis. Industry has relied heavily on building grouping hypotheses based only on similar structural and/or other physicochemical parameters, leading to a high rejection rate of REACH registration dossiers. Strengthening the group by incorporating a similarity assessment of mechanistic (eco)toxicological effects is a potential solution for reducing uncertainty in G/RAx, with the OECD stating that “*the most compelling evidence in support of a read-across hypothesis is information on a common mode of action of the substances and a mechanistic rationale for their common biological behaviour*” (OECD 2017). The question arises as to which experimental strategies could be used to generate the bridging data for the source and target substances, informing on the mode of action (MoA). Arguably, for data-poor targets, a broad range of potential MoAs should be examined to ensure that any significant toxicological effects are included in the similarity assessment. Therefore, ‘omics technologies, such as transcriptomics (i.e., systematic study of gene expression providing information on upstream pathway perturbations) and metabolomics (i.e., study of the levels of small-molecule endogenous metabolites, providing information on more downstream functional responses), are a highly relevant type of approach to apply as part of a bridging study.

This case study aimed to demonstrate how multi-omics measurements can improve the confidence in forming categories of data-poor chemicals, enabling the read-across of a toxicological endpoint to fill a data gap for a target substance. This work builds on earlier demonstrations of metabolomics and transcriptomics-based G/RAx (Grimm et al. 2019; Sperber et al. 2019; OECD 2020; Nakagawa et al. 2021; Vrijenhoek et al. 2022) by implementing and demonstrating the value of combining ‘omics methodologies for the ecotoxicological risk assessment of azo dyes. Disperse Yellow 3 (DY3) is the target substance and long-term toxicity to aquatic invertebrates (*Daphnia magna*) is the apical endpoint to be read across. Azo dyes are widely used in the textile industry and account for up to 70% of all dyestuff in textile production (Brüschweiler & Merlot 2017). Some have been shown to have negative impacts on human and environmental health, the former associated with the release of aromatic amines resulting in genotoxicity, mutagenicity and/or carcinogenicity (Chung 2016; Brüschweiler & Merlot 2017). Azo dyes can negatively impact aquatic health in the environment due to decreased light penetration and photosynthetic activity, resulting in oxygen deficiency and dysregulation of biological cycles (Przystaś, et al., 2012). Azo dyes have also been shown to disrupt microbial communities in soil, and to disrupt the germination and growth of plants (Lellis et al. 2019). Our first objective was to investigate six potential source substances (all azo dyes), applying conventional approaches to generate a grouping hypothesis based on structural similarity and quantitative structure–activity relationship (QSAR) profiling. The second objective was to apply multi-omics approaches (polar and apolar metabolomics, transcriptomics) to generate bioactivity profiles for each azo dye, measured in acute toxicity tests with *D. magna*, and then to calculate the ‘bioactivity similarity’ between the dyes. The metabolomics datasets were also analysed to seek evidence for internal exposure of *D. magna* to each azo dye. Quantifying the similarities of the bioactivity profiles generates an alternative grouping hypothesis, which may either substantiate or disprove the structure-based grouping hypothesis. The third objective was to map the structural fingerprints of the azo dyes onto the alternative grouping hypothesis to discover which structural features could be driving the bioactivity profile-based grouping. The fourth objective was to read across the endpoint, long-term toxicity to *Daphnia*, from the selected source substance to fill the data gap for DY3. The chronic toxicity of DY3 was measured experimentally to attempt to confirm the read-across prediction and thereby add confidence to this ‘omics bioactivity profile-based G/RAx workflow.

## Materials and Methods

### Azo dyes

Criteria were developed to guide the substance selection, including regulatory and experimental considerations (Online-Resource 1, Table S1). Seven azo dyes were selected comprising one target (Disperse Yellow 3, DY3) and six potential source substances; Sudan Red G (SRG), Sudan 1 (S1), Disperse Orange 25 (DO25), Disperse Orange 61 (DO61), Disperse Red 1 (DR1) and Disperse Red 13 (DR13). All dyes had a purity ≥ 95% and were purchased from LGC Standards (UK), except for DO25 purchased from Sigma-Aldrich (UK). Identifiers are provided in Online-Resource 1, Table S2 and the structures presented in Online-Resource 1, Fig. S1.

### Conventional grouping approaches

Experimental (where available) and predicted physico-chemical properties for the azo dyes are provided in Online-Resource 1, Table S3. (Q)SAR alerts based on chemical fragments were collected for each azo dye using several profilers in the OECD QSAR Toolbox (v4.3), including ECOlogical Structure Activity Relationships (ECOSAR v2.0) classification and OASIS acute toxicity mode of action. A conventional grouping hypothesis was formed by manually examining these (Q)SAR alerts. Confidence in this grouping was increased by calculating the structural similarities between the dyes based on Tanimoto coefficients, representing each dye by a ToxPrint chemotype (729 bits encoding physicochemical properties of atoms, bonds and structural fragments). Using the *pvclust* package (version 2.2–0; Suzuki & Shimodaira 2006), the binary distance (method.dist = "binary") was calculated between every pair of dyes, which were assimilated into a distance matrix for all dyes and hierarchically clustered (method.hclust = "ward.D2") with multiscale bootstrap resampling (n = 10,000 bootstrap pseudo-replications). Clusters where the pvclust calculated selective inference (SI) value was ≥ 0.95, were strongly supported by the data.

### *Daphnia magna* exposure studies

The overall workflow for the acute *D. magna* toxicity studies to generate samples for the multi-omics measurements is shown in Online-Resource 1, Fig. S2, and described in detail in Online-Resource 1, Section S1.1, comprising acute dose range-finding, benchmark dose modelling and further acute exposures. In brief, acute (48-h) toxicity of the azo dyes was determined following OECD test guidelines (OECD 2004) after consulting published data to establish crude dose ranges, using ≥ 5 nominal doses of each dye (Online-Resource 1, Tables S4–S5). Dimethyl sulfoxide (DMSO) was used as a carrier solvent for all samples (final concentration 0.1%). Benchmark dose (BMD) modelling was conducted on 48-h immobilisation data using PROAST, exporting lower and upper estimates of BMD (BMDL and BMDU, respectively), from which doses for the ‘omics study were derived. The multi-omics experimental design included three sampling time points (2-h, 24-h and 48-h) and three dose groups plus untreated controls (0.1% DMSO) per dye, which were conducted in six exposure batches (Online-Resource 1, Table S6). Doses were phenotypically anchored to 10% *Daphnia* immobilisation (‘high’ dose), from the BMD modelling (i.e. CES of 0.1); ‘medium’ and ‘low’ doses were sequentially half-log_10_ lower due to the steepness of the dose–response curves. Each replicate sample for ‘omics analysis contained ten to twelve *D. magna* (< 24-h old) per exposure vessel, with n = 6 vessels per treatment group. At each sampling time point, *D. magna* from each exposure vessel were divided equally for metabolomics and transcriptomics (each containing 5–6 animals per replicate sample). Samples were collected for ‘omics, flash-frozen in liquid nitrogen and stored at − 80 °C. *D. magna* chronic (21-d) toxicity was determined per OECD test guidelines (OECD 2012) and described in Online-Resource 1, Section S1.2.

### Polar and apolar metabolomics: sample extraction, data acquisition, processing and feature annotations

Detailed methods are described in Online-Resource 1, Section S1.3. In brief, polar and apolar metabolites were extracted from frozen *D. magna* and separately analysed by nanoelectrospray direct infusion mass spectrometry (DIMS) metabolomics, as reported previously (Southam et al. 2017). The MEtabolomics standaRds Initiative in Toxicology (MERIT) best practice guidelines (Viant et al. 2019) were followed, including use of a system suitability quality control (QC), intrastudy QC and process blank samples. Data acquisition was conducted using an Orbitrap Elite mass spectrometer (Thermo Scientific) interfaced with a Triversa NanoMate nanoelectrospray source (Advion). DIMS data were processed using DIMSPy, implemented in Galaxy, including steps to remove any dye-related features from the endogenous feature matrices prior to statistical analysis. The processed data matrices of feature intensities from the polar and apolar DIMS assays are provided in Online-Resources 2 and 3, respectively. Features were putatively annotated using the Birmingham mEtabolite Annotation for Mass Spectrometry (BEAMS) pipeline (https://more.bham.ac.uk/beams/).

### Untargeted xenobiotic analysis

An untargeted xenobiotic data analysis workflow was applied to each of the DIMS metabolomics feature matrices, as described previously (Bowen et al. 2023) with slight modifications, to attempt to confirm the internal exposure of *D. magna* to each azo dye and to discover metabolic biotransformation products (BTPs). The workflow was applied to each ‘blank-filtered’ feature matrix, with settings to reject all endogenous biochemicals, as detailed in Online-Resource 1, Section S1.4. Dye-related *m/z* features were putatively annotated by matching to a list of in silico predicted phase I and II BTPs (Systematic Generation of potential Metabolites, SyGMa; (Ridder & Wagener 2008)) for each azo dye. Confidence in these annotations was increased when their normalised intensities were observed to correlate with the nominal exposure doses of the azo dyes.

### Transcriptomics: RNA extraction, data acquisition, processing and gene annotations

Detailed methods are described in Online-Resource 1, Section S1.5. In brief, total RNA was extracted from frozen *Daphnia* samples using the Agencourt RNAdvance Tissue Kit (Beckman Coulter); only n = 4 replicate samples were analysed per treatment group. Expression data were obtained using TempO-Seq® and a custom BioSpyder platform consisting of 1991 *D. magna* genes (Online-Resource 4, Table S7). This probe set contains *Daphnia* genes with homology to human genes from the Molecular Signature Database and S1500 + gene set in addition to a subset identified during a pilot *D. magna* exposure to DR1 (data not shown). Raw counts were summarised to gene level and genes with low counts (< 10 across all samples) were removed. Count normalisation and differential expression analysis were conducted in R using the *DESeq2* package. The processed data matrix of gene counts from the transcriptomics assay is provided in Online-Resource 5. Analyses were performed on treated and control samples for each dye separately, treating each dose/time combination as a separate sample type (n = 4).

### Statistical analyses to group substances using single and multi-omics data

Following standard processing of the three ‘omics data streams described above, the transcriptomics, DIMS polar and apolar metabolomics were grouped using the workflow summarised in Online-Resource 1, Fig. S3. All statistical analyses were conducted in the R environment (version R-4.0.3), using the *structToolbox* (version 1.3.1; Lloyd et al. 2021) *DESeq2* (version 1.30.0; Love et al. 2014) and *pvclust* packages (version 2.2–0; Suzuki & Shimodaira 2006). First, principal component analysis (PCA) was applied to identify any outlying samples in each ‘omics dataset for each dye, with samples lying outside a 95% confidence limit being removed. Next, independent two-sample t-tests were applied across all molecular features (genes and *m/z* features), comparing dye-specific untreated controls with each of nine treated dose/time groups (per dye), to gain insights into the magnitude of perturbations induced by each treatment. Also, these t-statistics served as the basis for unsupervised hierarchical cluster analysis (HCA) to visualise the similarities of the ‘omics bioactivity profiles between treatments. Specifically, HCA was performed on the vector normalised (i.e., convert to a unit vector on a feature-by-feature basis) t-statistics (with n = 10,000 multiscale bootstrap pseudo-replications), utilising Euclidean distance and Ward’s linkage method (“ward.D2” method; Murtagh & Legendre 2014). HCA was applied to three different combinations of input data: t-statistics derived from the polar and apolar metabolomic responses only, transcriptomic responses only and a concatenated matrix of t-statistics from all three ‘omics datasets. In addition, for each of these combinations of input data, two further approaches were applied: first, HCA of all (typically 9) dose/time groups per dye, for all 7 dyes; and secondly, HCA of 3 dose groups per dye, for all 7 dyes, collapsing the time points to a single point by selecting only the largest transcriptional and largest metabolic perturbations (i.e., using the highest absolute t-statistic across the 3 time points, referred to as the ‘maximum-perturbation’ approach). Collectively, these HCA analyses enabled comparisons of single- versus multi-omics-based grouping; an overview of the effects of dose and time per dye, yielding relatively complex dendrograms; and more interpretable clustering of the seven dyes at low, medium and high doses.

## Results and discussion

### Establish a grouping hypothesis based on structural similarity and QSAR profiling

#### Define grouping/read-across scenario and assimilate existing data

This study corresponds to an ECHA RAAF scenario 2: Analogue approach, for which the read-across hypothesis is based on different compounds with qualitatively similar properties. The target is DY3, and we sought to use new and existing data to determine the most suitable analogue from a pool of six structurally similar azo dyes. The toxicological endpoint to be read across from source to target is long-term toxicity to aquatic invertebrates. First, all seven dyes’ chemical structures, purity profiles and physico-chemical properties were collected (Online-Resource 1, Fig. S1, Tables S2–S3, respectively). All the dyes comprise an azo functional group and substituted aromatic rings, providing an initial structure-based justification for the group. Since the reported purity for each dye is ≥ 95.0%, it was assumed that impurities do not contribute significantly to any toxicological effects observed in this study. A comparison of the physico-chemical properties is described in Online-Resource 1, Table S3 and Section S2.1. Existing aquatic toxicity data for the seven azo dyes are summarised in Online-Resource 1, Table S4. The NOEC and LOEC chronic aquatic toxicity for S1, DY3 and DO61 was measured and reported as part of this study (Online-Resource 1, Table S4).

#### Conventional grouping hypothesis based on structural similarity and QSAR profiling

**Fig. 1:**
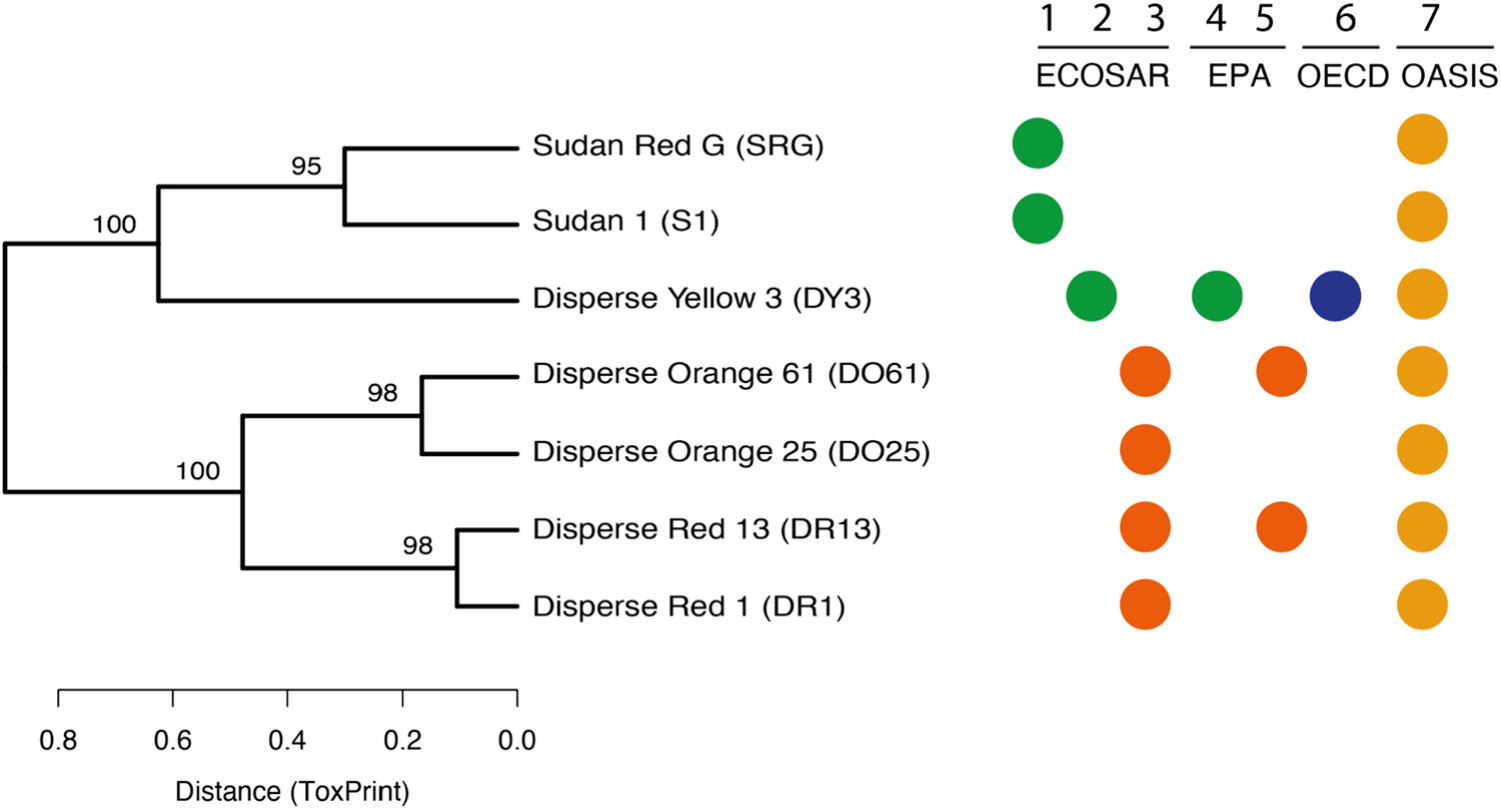
Structural similarity of the seven azo dyes derived from hierarchical cluster analysis of the binary distance calculated between each pair of dyes, using 33 non-zero structural fragments of ToxPrint chemotypes to encode the structures. Selective inference (SI) bootstrap replicability confidence values are shown at each node when ≥ 95%, indicating the dyes form two distinct groups: (1) DY3, S1, SRG, and (2) DO25, DO61, DR1, DR13. (Q)SAR profiler alerts are shown for each substance obtained from ECOSAR 2.0 (ECOSAR), US EPA New Chemical Categories (EPA), OECD HPV Chemical Categories (OECD) and OASIS as follows: (1) Belongs to phenols, (2) Belongs to phenols, amides & phenol amines, (3) Belongs to neutral organics, (4) Belongs to phenols (acute toxicity), (5) Belongs to neutral organics, (6) Belongs to m,p-cresols, (7) Reactive unspecified alert by acute aquatic toxicity. The groups are colour-coded according to analogous profile descriptors across different profilers: green classifies phenols, dark orange classifies neutral organics, blue classifies m,p-cresols, yellow classifies reactive unspecified alert by acute aquatic toxicity mode of action

The seven azo dyes were grouped using structural similarity (ToxPrint chemotypes) and (Q)SAR profiling to formulate a conventional grouping hypothesis, against which the ‘omics bioactivity profile-based grouping would be compared. The grouping presented here uses hierarchical cluster analysis (HCA), ensuring consistency across the conventional and ‘omics approaches, visualising group membership in dendrograms. HCA groups substances based on the distances between their underlying data, and is widely used to analyse high-dimensional datasets. First, we applied HCA to partition the seven dyes into categories based on chemical structure. Structure-based grouping using ToxPrint chemotypes (Fig. 1) reveals two distinct groups of dyes: (1) S1, SRG and the target DY3 and (2) DR1, DR13, DO25 and DO61. Therefore, we initially concluded that the two Sudan dyes are the more suitable analogues for the read-across to DY3. Next, multiple (Q)SAR profilers relevant to aquatic toxicity were applied using the (Q)SAR Toolbox (v4.3), including aquatic toxicity classification by ECOSAR, acute aquatic toxicity MOA by OASIS, OECD HPV Chemical Categories and US-EPA New Chemical Categories (Fig. 1). These results indicate that the seven dyes may be classified into three groups: (1) DY3, (2) Sudan dyes and (3) disperse orange and red dyes. However, the ECOSAR profiler identifies both DY3 and the Sudan dyes as belonging to phenols, suggesting that these three dyes (including the target) may form one group, which would be more consistent with the findings from the purely structural comparison. In conclusion, applying conventional approaches generates a grouping hypothesis with the target, DY3, most likely belonging to the same small group as the two Sudan dyes, though with some uncertainty.

### Determine an alternative grouping hypothesis based on bioactivity similarity using multi-omics data

#### Acute aquatic toxicity of azo dyes to *Daphnia*

First, 48-h dose range-finding studies were conducted with *Daphnia* and benchmark dose modelling applied to determine the nominal aqueous concentrations that induced 10% immobilisation, referred to as the ‘equi-effective dose’ for each dye (Online-Resource 1, Table S5). As DO25 did not immobilise the *Daphnia* at any dose investigated, its equi-effective dose was set to the highest equi-effective dose across the six other dyes. The equi-effective doses were used for two purposes: (i) to determine the relative potencies of the dyes to ensure the ‘worst-case approach’ criteria were met for the read-across (Sect. "Read-across apical endpoint and confirm the prediction experimentally"), and (ii) to define the three nominal exposure concentrations, per dye, used to generate samples for multi-omics measurements (Online-Resource 1, Table S6). The ‘omics exposure study comprised three doses (‘low’, ‘medium’ and ‘high’, plus untreated controls) and three sampling time points (2-, 24- and 48-h), hence nine dose/time groups per dye, and a total of 63 treatment groups in the study. Two 48-h groups were discarded for S1 (medium and high doses) and one for DO61 (high dose) due to high *Daphnia* immobilisation, reducing the total number of groups for multi-omics measurements to 60.

#### Untargeted xenobiotic analysis confirms azo dye internal exposure and metabolism

Polar and apolar metabolomics data were generated as described in Sect. "Polar and apolar metabolomics: sample extraction, data acquisition, processing and feature annotations", primarily to detect endogenous biochemical perturbations, but were also analysed using an untargeted xenobiotic workflow to seek confirmation of internal exposure of *D. magna* to each azo dye, and to attempt to discover any metabolic BTPs. All seven dyes were detected in the *Daphnia* extracts (Table 1), predominantly in the apolar DIMS dataset, consistent with their high log K_ow_ values (Online-Resource 1, Table S3). The relative intensities of several of the dyes increased across the three nominal exposure doses, confirming dose-dependent internal exposure of the *Daphnia* (Online-Resource 1, Fig. S4). In addition, the untargeted xenobiotic workflow discovered BTPs for the target DY3 and potential analogues S1, SRG and DO25, summarised in Table 1 and detailed in Online-Resource 1, Tables S8 and S9. While these observations provided evidence for internal exposure to each dye, incorporating the biotransformation data into the grouping hypothesis was beyond the scope of this paper. However, the potentially considerable added value of these data for supporting a grouping hypothesis by providing evidence of shared metabolism (i.e. shared BTPs between target and source substances) should be noted.

**Table 1:** Azo dye parent and metabolic biotransformation products detected and putatively annotated in the DIMS polar and apolar metabolomics datasets

	Extract measured by DIMS metabolomics	DY3	S1	SRG	DR1	DR13	DO25	DO61
Parent chemical detected	Apolar	Yes	Yes	Yes	Yes	Yes	Yes*	Yes
	Polar	Yes	Yes	Yes*	Yes	No	Yes	No
No. of BTPs detected	Apolar	4	1	2	0	0	1*	0
	Polar	2	0	1	0	0	0	0

*Tentative annotation as fold-change filtering criteria were not met

#### *Daphnia* multi-omics bridging study of azo dyes

**Fig. 2:**
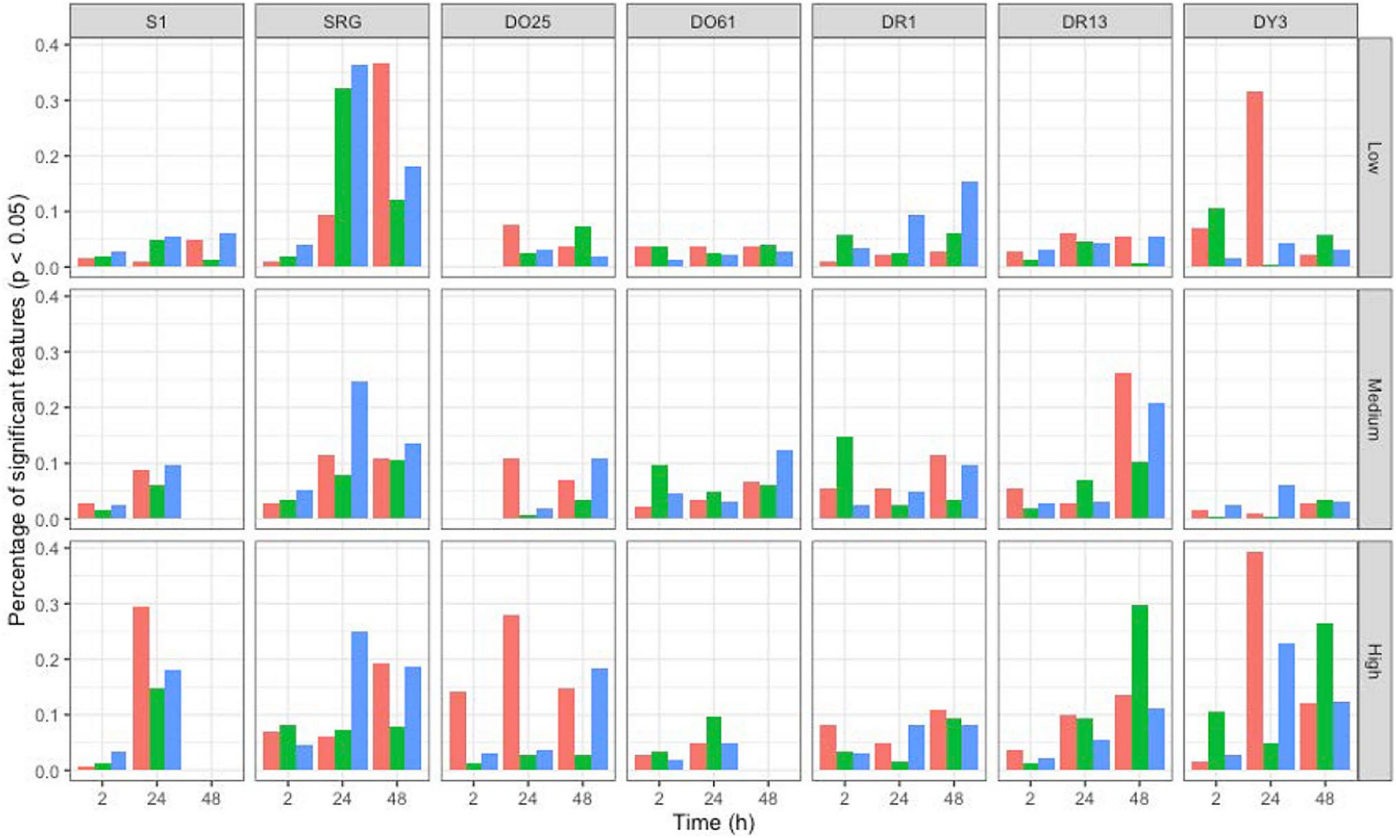
Bar charts showing proportion of the total number of features detected within each ‘omics dataset (apolar metabolomics—red; polar metabolomics—green; transcriptomics—blue) that are differentially abundant (*p* < 0.05) between treated and control samples of *Daphnia magna* neonates collected following 2-, 24- and 48-h exposures to low (top panel), medium (mid panel) and high doses (bottom panel) of seven azo dyes (S1, SRG, DO25, DO61, DR1, DR13, DY3)

Polar and apolar metabolomics (both using direct infusion mass spectrometry) and transcriptomics (using the BioSpyder TempO-Seq® 1991-gene array) responses were measured as described in Sects. "Polar and apolar metabolomics: sample extraction, data acquisition, processing and feature annotations" and "Transcriptomics: RNA extraction, data acquisition, processing and gene annotations", primarily to detect endogenous molecular perturbations induced by exposure to each dye, at three doses and three time points. Each of the large-scale raw datasets (e.g., a total of 2,204 DIMS analyses were conducted on 760 *Daphnia* samples) was processed individually, generating a positive ion DIMS polar metabolomics dataset (referred to below as ‘polar metabolomics’; 245 features, median relative standard deviation (RSD) of intrastudy QC samples of 26.6%), a positive ion DIMS apolar dataset (‘apolar metabolomics’; 183 features, median RSD of intrastudy QC samples of 21.8%) and a transcriptomics dataset (1889 genes after processing). Of the 63 possible treatment groups (7 dyes, 3 doses, 3 time points), three were not measured due to high *Daphnia* immobilisation (48-h medium and high doses for S1, 48-h high dose for DO61). Two were removed by quality-filtering during data processing and PCA (2-h low- and medium-dose groups for DO25), leaving 58 dose/time groups for statistical analysis; note that due to the integrative multi-omics analyses conducted, if ‘omics data were missing for one data stream, that treatment group was removed from all further analyses. Figure 2 confirms that polar metabolic, apolar metabolic and transcriptional changes were induced by exposure to the azo dyes, represented graphically as the percentage of features that changed significantly (*p* < 0.05) per assay, for each of 58 treatment groups. In general, a greater percentage of features demonstrated significant changes at the later 24- and 48-h time points than at 2-h, with a similar lower level of response in low-dose samples versus medium and high concentrations.

#### Bioactivity similarity of azo dyes using multi-omics molecular data

The bioactivity similarity of the seven azo dyes was calculated and visualised to determine which of the six potential source substances exhibits a molecular effect that most closely resembles the response of *D. magna* to the target, DY3. Hierarchical cluster analysis (HCA) was performed initially on all 58 remaining treatment groups (all dyes, doses and time points), using the DIMS polar and apolar metabolomics data (Online-Resource 1, Fig. S5a), transcriptomics data (Fig. S5b) and all three ‘omics datasets combined (Fig. S5c). One anticipated pattern observed was the grouping of low-, medium- and high-dose groups (which span a concentration range of less than one order of magnitude), conditional on the time points, suggesting that the ‘omics workflow is achieving its intended purpose of grouping similar molecular responses. This pattern was particularly evident when all three datasets were combined, with 14 of the 18 remaining dye/time treatments demonstrating low, medium and high doses clustered together.

**Fig. 3:**
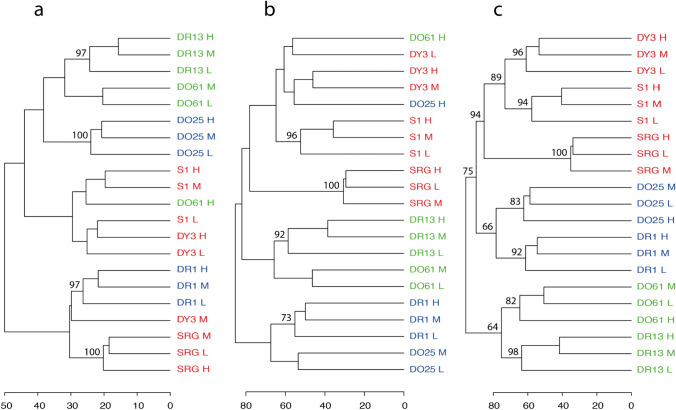
Dendrograms produced by the HCA grouping workflow using t-statistics derived from **a** polar and apolar metabolomics data comprising 428 features, **b** transcriptomics data comprising 1889 features and **c** all three ‘omics datasets combined, from samples of *Daphnia magna* neonates collected at the exposure time producing the maximum biomolecular perturbations at low, medium and high doses of seven azo dyes (DY3, S1, SRG, DR1, DR13, DO25, DO61), corresponding to 21 treatment groups. X-axis values indicate the distance measurements (the sum of branch lengths) among any pair of doses and substances. The values at the top of the branches indicate % bootstrap replicability confidence (using approximately unbiased (AU) tests of non-selective inference) for nodes that are grouping all three doses for the same substance (for a-c), and confidence in the ‘omics grouping of the seven dyes substantiating the hypothesis derived from the structure-based grouping shown in Fig. 1 (shown in c only). The labels on all three dendrograms are coloured according to the membership of the seven azo dyes within three multi-omics defined groups (panel c: red, blue, green) to facilitate comparisons with single-omics grouping (panels a, b)

Next, an approach was implemented to reduce the complexity of the visualisation, reducing the 2-h, 24-h and 48-h data to a single time point by comparing and then selecting only the largest transcriptional and largest metabolic perturbations, referred to as the maximum-perturbation approach (Sect. "Statistical analyses to group substances using single and multi-omics data"). HCA was performed on this reduced 21-treatment group dataset using the DIMS polar and apolar metabolomics (Fig. 3a), transcriptomics data (Fig. 3b) and all three ‘omics datasets combined (Fig. 3c). Again, the clustering of the low, medium and high doses for a majority of dyes is evident when selected ‘omics modalities are used and is observed for all seven dyes when analysing the combined ‘omics data. Overall, a higher confidence in the grouping pattern (i.e., occurrence of more high bootstrap replicability confidence values of > 80% using approximately unbiased (AU) tests of non-selective inference) is achieved when analysing the three concatenated ‘omics datasets. This demonstrates value in combining a range of upstream and downstream molecular changes into the bioactivity similarity statistical assessment. Of principal interest is which source substance groups closest to the target. Grouping based on chemical structures (Fig. 1) indicated that S1 and SRG were equally similar to DY3, forming a distinct group separated from the remaining four dyes. This structure-based grouping is strongly supported by the bioactivity profile-based grouping (94% bootstrap replicability confidence, i.e., 6% probability that the grouping is not true) despite a small ratio of the average distance between this group (DY3, S1, SRG) and the neighbouring group (DO25, DR1) over the average intergroup distances (Fig. 3c). Furthermore, bioactivity profile-based grouping indicated that S1 is more similar to the target DY3 than SRG (89% bootstrap replicability confidence using AU tests of non-selective inference). By contrast, among the 10,000 pseudo-replicates, no dendrograms were found to have an equivalent cluster where all dose groups of DY3 and SRG are grouped into a single cluster at the exclusion of other substances. Therefore, we conclude that, based on a statistical assessment the ‘omics data have substantiated the grouping hypothesis derived using chemical structure, confirming that S1 and SRG are the only valid analogues. Additionally, the ‘omics data uniquely revealed that the bioactivity of S1 is most similar to the target and therefore this dye is selected as the source for read-across to DY3.

### Identify structural features that may drive the alternative grouping hypothesis

**Fig. 4:**
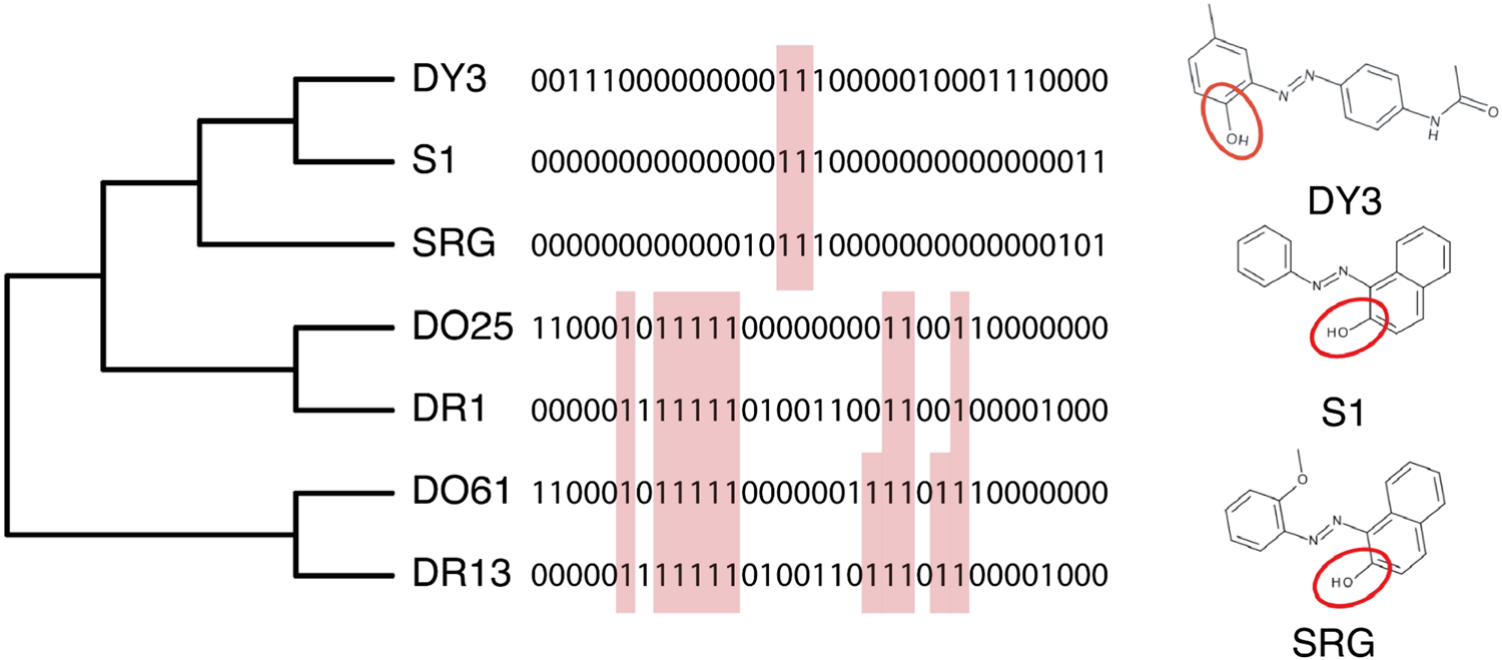
Mapping of the 33 non-zero structural fragments of ToxPrint chemotypes onto the dendrogram derived from multi-omics bioactivity profile-based grouping (Fig. 3). Red shading indicates the non-zero structural fragments that are shared within a substance group. Only two aromatic alcohol fragments are unique to the DY3, S1 and SRG group, which are depicted for all three azo dyes: COH alcohol aromatic bond, COH alcohol aromatic phenol bond

Structural fingerprints as ToxPrint chemotypes of the azo dyes were mapped to the alternative grouping hypothesis (derived from the bioactivity similarity assessment, shown in Fig. 3) to detect which structural features could be driving the bioactivity profile-based grouping. The aim was to strengthen the grouping hypothesis by associating the structural and biological elements. ToxPrints consist of a binary encoding of 729 structural fragments (e.g. atomic, bond and chain types) associated with biological properties and modes of action (Yang et al. 2015). Using the non-zero structural fragments (i.e., those fragments that are present in a dye) of the ToxPrints chemotypes that are shared within substance groups (red shading, Fig. 4), only two aromatic alcohol fragments are unique to the group comprising DY3, S1 and SRG. This finding suggests that the aromatic alcohol moiety could be responsible for driving the ‘omics responses observed in *D. magna*. A more extensive analysis for all 7 dyes is presented in Online-Resource 1, Table S10.

### Read-across apical endpoint and confirm the prediction experimentally

**Fig. 5:**
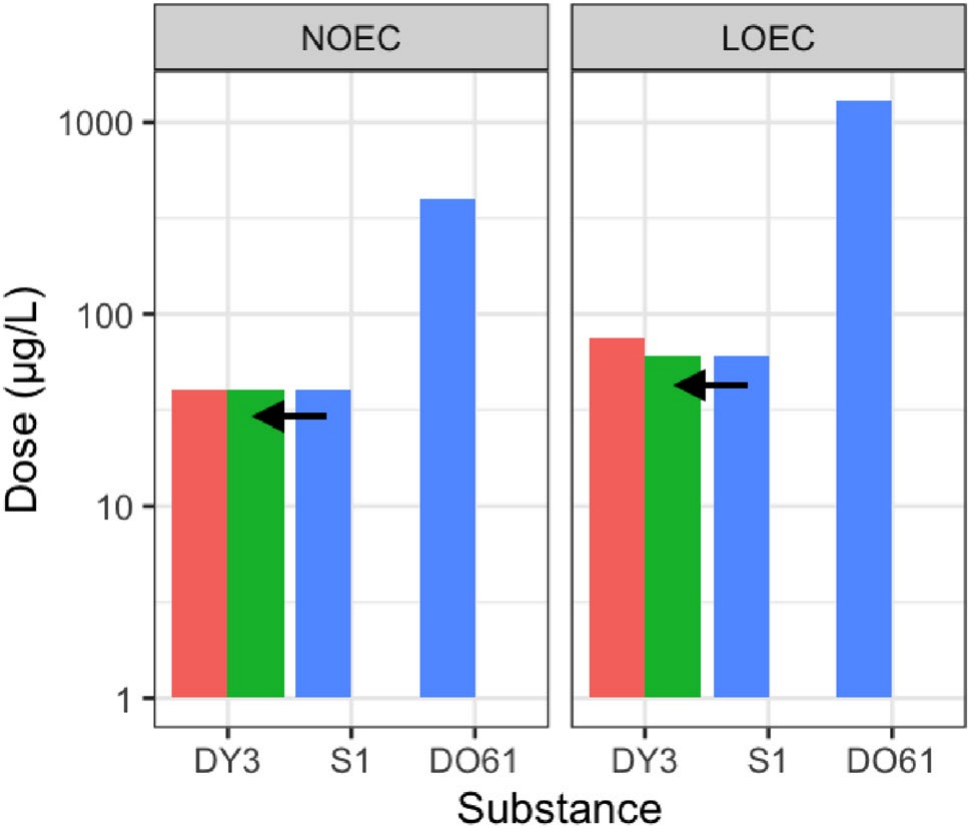
Chronic reproductive toxicity to *Daphnia magna* of the target Disperse Yellow 3 (DY3)*,* showing the accuracy of the predicted (green) no-observed-effect-concentration (NOEC) and lowest-observed-effect-concentration (LOEC) values—derived by reading across (black arrows) the measured toxicity values from the source Sudan 1 (S1; blue)—with the experimentally measured values for DY3 (red). Measured toxicity values for Disperse Orange 61 (DO61; blue), which was shown to induce *dissimilar* ‘omics responses to DY3, are also illustrated

Given the structure-based grouping proposed S1 and SRG as potential source substances, and the ‘omics bioactivity profile-based grouping uniquely demonstrated that S1 is the most suitable analogue for read-across to DY3, we then predicted the *D. magna* chronic reproductive toxicity of the target; i.e., we read across a 21 days NOEC of 40 µgL^−1^ and LOEC of 60 µgL^−1^ from S1 (Online-Resource 1, Table S4) to DY3. This meets the criteria in the RAAF for a conservative prediction (the ‘worst-case approach’, reading from a more potent to less potent substance) as the potency of S1 is higher than for DY3 (based upon the measured *D. magna* acute toxicity). To confirm this read-across prediction and add confidence to the bioactivity profile-based grouping/read-across workflow presented here, the chronic toxicity of DY3 was measured experimentally in *D. magna*. Although the predicted log K_ow_ values for these two substances differ somewhat (Table S3), suggesting some difference in toxicity, the measured values were similar to the predicted 21 days NOEC and LOEC for DY3 (Fig. 5). To add further confidence to the bioactivity profile-based grouping results, the chronic toxicity of DO61, which groups away from DY3, was considered. The measured toxicity for DO61 was much lower than DY3, confirming that it would not be an appropriate source substance (Fig. 5). Together these results demonstrate that the ‘omics bioactivity profile-based grouping can improve the confidence in analogue selection for data-poor substances.

### Limitations of multi-omics workflow for chemical grouping

While this study successfully demonstrated how multi-omics technologies can add value to a chemical grouping workflow, limitations were identified. The most major concern is the evidence generated for the analogue justification. Undeniably, DY3 (target) and S1 (source) do share common structural features (Fig. 1) and also exhibit quantitatively similar ‘omics response profiles confirmed using statistical approaches with associated probabilities (Fig. 3c). This,in turn, *suggests* these dyes share a similar MoA. However, what is lacking from this study is a plausible toxicological interpretation of the molecular data that could provide a third layer of evidence, which together with the structural similarity and ‘omics-based bioactivity profile-based grouping could form a stronger analogue justification. There are two reasons we did not attempt a toxicological interpretation of the ‘omics data to support the read-across primarily, the metabolomics data were derived using DIMS for which it is difficult to identify metabolites with high confidence, hence a reliable biochemical interpretation of that ‘omics data was not feasible. Furthermore, the transcriptomics data were measured using a reduced gene set, which limited our ability to conduct an analysis of known functional pathways that are enriched by the signature gene set. Given this case study was focused on demonstrating high confidence in the use of ‘omics-derived bioactivity similarities for grouping, we concluded it was not appropriate to use low-confidence methods to attempt any toxicological interpretation. We recommend that future case studies should employ (1) genome-wide transcriptomics and (2) hybrid liquid chromatography–mass spectrometry metabolomics, which in addition to untargeted profiling can also target pre-selected metabolic biomarkers, as we have recently proposed (MTox700 + biomarker list; Sostare et al. 2022). Together, this would increase confidence in the metabolite identification and enable multi-omics pathway analysis to help provide a plausible toxicological interpretation for a chemical group.

There are two additional limitations when considering the regulatory context. These include the lack of reporting of this case study using internationally accepted guidelines for ‘omics bioactivity profile-based G/RAx, and the use of approaches that have not yet been validated, including the unproven reliability of bioactivity profile-based grouping using ‘omics data. Regarding the former, the OECD Omics Reporting Framework (OORF) has recently been developed to provide guidance on reporting the acquisition, processing and statistical analysis of ‘omics data in regulatory toxicology (Harrill et al. 2021). Additionally, a project by the OECD Working Party on Hazard Assessment is currently defining how to report chemical grouping using ‘omics data. Regarding a lack of validation, the reliability of chemical grouping using metabolomics data has recently been demonstrated in the Cefic LRI-funded MATCHING (MetAbolomics ring-Trial for CHemical groupING) project (Viant et al. 2024). This ring-trial comprised six blinded laboratories each acquiring, processing and statistically analysing a set of rat plasma samples obtained from a 28-day exposure study, with five partners achieving an identical grouping of eight test substances. Also, a new framework has just been proposed to evaluate the quality and reliability of targeted metabolomics assays, including in toxicology (Sarmad et al., 2023). These recent and ongoing efforts all contribute significantly to building confidence in the application of ‘omics data to regulatory toxicology.

While not a limitation of the multi-omics workflow for chemical grouping per se, this case study provides an example where the results derived from bioactivity profile-based grouping, structural and QSAR approaches are all in agreement. Thus yielding a consistent hypothesis for the selection of an analogue to the DY3 target substance. Currently, there is no international guidance on how to reconcile differing hypotheses from biological-based grouping and structural approaches for chemical grouping: this situation is likely to arise in future studies. Further well-designed case studies are needed to evaluate and determine where the precedence should lie, i.e., how to weigh each approach’s relative contributions to the final grouping hypothesis.

## Conclusions

We have demonstrated how metabolomics and transcriptomics can be combined to improve the confidence in grouping data-poor chemicals, enabling the read-across of a toxicological endpoint to fill a data gap for a target substance. Multi-omics bioactivity profile-based grouping uniquely revealed that S1 is the most suitable analogue for read-across to DY3, supported by statistical evidence, and substantiates the structure-based grouping using (Q)SAR profilers that identified S1, SRG and DY3 as a group. The read-across of *Daphnia* chronic reproductive toxicity from S1 to DY3 was subsequently confirmed experimentally, increasing confidence in the capability of the multi-omics workflow. Mapping ToxPrint structural fingerprints of the dyes onto the bioactivity profile-based grouping indicated an aromatic alcohol moiety could be responsible for the shared molecular effects of S1 and DY3. Additionally, the metabolomics measurements provided insights into ADME processes, enabling the simultaneous discovery of the fate and biochemical effects of the azo dyes. The ‘omics measurements confirmed internal exposures of *D. magna* to each dye and discovered multiple biotransformation products. Some limitations of the overall approach have been identified. Foremost is the lack of plausible toxicological interpretation of the molecular effects, which was not feasible for technical reasons yet could in principle provide a third layer of evidence for the analogue justification, building on the structural similarity and bioactivity similarity of the ‘omics profiles. A further limitation related to the future regulatory application of bioactivity profile-based grouping is the lack of standardised methodologies and reporting. However, efforts are underway to help address these issues. While acknowledging these limitations, this case study demonstrates an effective multi-omics NAM for building chemical groups to enable read-across.

### Supplementary Information

Below is the link to the electronic supplementary material.

Supplementary file1 (DOCX 1019 KB)

Supplementary file2 (XLSX 4967 KB)

Supplementary file3 (XLSX 3836 KB)

Supplementary file4 (XLSX 468 KB)

Supplementary file5 (XLSX 3306 KB)

## Data Availability

All data generated during this study are included in this published article and its supplementary information files.
