# Supplementary material for: Multi-omics bioactivity profile-based chemical grouping and read-across: a case study with Daphnia magna and azo dyes
Source: Arch Toxicol. 2024 May 2;98(8):2577–88. doi: 10.1007/s00204-024-03759-6 (PMC11272716; doi:10.1007/s00204-024-03759-6)
Supplement: Supplementary file 1 — Supplementary file1 (DOCX 1019 KB) [file 204_2024_3759_MOESM1_ESM.docx]

# Supplementary Information

# Archives in Toxicology

# Multi-omics bioactivity profile-based chemical grouping and read-across: A case study with *Daphnia magna* and azo dyes

Hanna Gruszczynska ^a^, Rosemary E. Barnett ^b^, Gavin R. Lloyd ^c^, Ralf J. M. Weber ^a,c^, Thomas N. Lawson ^b^, Jiarui Zhou ^a^, Elena Sostare ^b^, John K. Colbourne ^a,b^, Mark R. Viant ^a,b,c,*^

^a^ School of Biosciences, University of Birmingham, Edgbaston, Birmingham, B15 2TT, United Kingdom

^b^ Michabo Health Science Limited, Union House, 111 New Union Street, Coventry CV1 2NT, United Kingdom

^c^ Phenome Centre Birmingham, University of Birmingham, Edgbaston, Birmingham, B15 2TT, United Kingdom

* Corresponding author (Mark R. Viant)

E-mail: [mark@michabo.co.uk](mailto:mark@michabo.co.uk)

Address: Michabo Health Science Limited, Union House, 111 New Union Street, Coventry CV1 2NT, United Kingdom

ORCID: <https://orcid.org/0000-0001-5898-4119>

## 1. Materials and Methods

**Table S1.** Summary of criteria guiding the selection of azo dyes for the grouping study.

| **Regulatory considerations** | **Experimental considerations** |
| --- | --- |
| 1. Structure: a specific core and different functional groups (no homologous series)  2. *Daphnia* acute toxicity data (OECD Test Guideline 202): available or predicted  3. *Daphnia* chronic toxicity data (OECD Test Guideline 211): available or predicted  4. Include REACH registered substances | 1. Available to purchase  2. Available at high purity (≥95%)  3. At least partial water solubility with low volatility; i.e. log 𝖪_ow_ (n-octanol/water partition coefficient) of approx. 4–7 |

**Table S2.** Azo dye identifiers, including Chemical Abstracts Services (CAS) registry number, European Community (EC) number, and Simplified Molecular-Input Line-Entry System (SMILES) notation; and commercial suppliers with purity.

| **Azo dye** | **Abbrev.** | **CAS-RN** | **EC** | **SMILES** | **Supplier**  **(batch no.)** | **Purity** |
| --- | --- | --- | --- | --- | --- | --- |
| Sudan 1 | S1 | 842-07-9 | 212-668-2 | Oc(ccc(c1ccc2)c2)c1N=Nc(cccc3)c3 | LGC Standards (G170606) | 99.6% |
| Sudan Red G | SRG | 1229-55-6 | 214-968-9 | O(c(c(N=Nc(c(c(ccc1)cc2)c1)c2O)ccc3)c3)C | LGC Standards (G165933) | 97.1% |
| Disperse Orange 25 | DO25 | 31482-56-1 | 250-654-8 | N(=O)(=O)c(ccc(N=Nc(ccc(N(CCC(#N))CC)c1)c1)c2)c2 | Sigma-Aldrich (12106HB) | 95% |
| Disperse Orange 61 | DO61 | 55281-26-0 | 259-563-8 | N(=O)(=O)c(cc(c(N=Nc(ccc(N(CCC(#N))CC)c1)c1)c2Br)Br)c2 | LGC Standards (G167394) | 98.7% |
| Disperse Red 1 | DR1 | 2872-52-8 | 220-704-3 | O=N(=O)c(ccc(N=Nc(ccc(N(CCO)CC)c1)c1)c2)c2 | LGC Standards (G132128) | 98.3% |
| Disperse Red 13 | DR13 | 3180-81-2 | 221-688-1 | O=N(=O)c(ccc(N=Nc(ccc(N(CCO)CC)c1)c1)c2Cl)c2 | LGC Standards (737819) | 99.2% |
| Disperse Yellow 3 | DY3 | 2832-40-8 | 220-600-8 | O=C(Nc(ccc(N=Nc(c(O)ccc1C)c1)c2)c2)C | LGC Standards (G122504) | 98.8% |


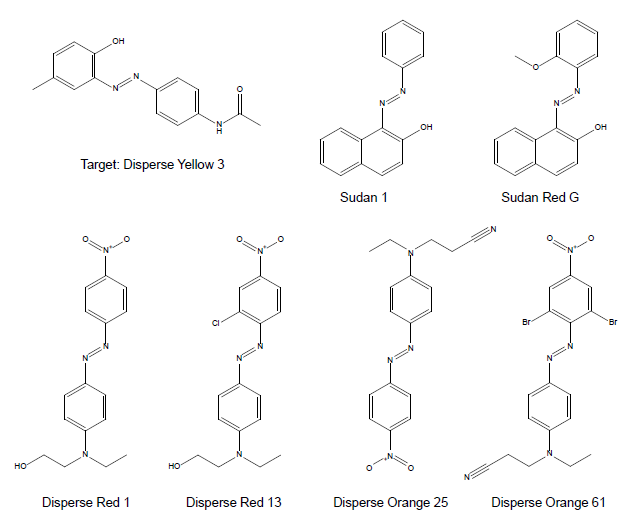


**Fig. S1** Azo dye chemical structures: Sudan 1 (S1), Sudan Red G (SRG), Disperse Orange 25 (DO25), Disperse Orange 61 (DO61), Disperse Red 1 (DR1), Disperse Red 13 (DR13) and the target substance, Disperse Yellow 3 (DY3)

**Table S3.** Summary of physico-chemical properties and organic functional groups of the seven azo dyes investigated: Sudan 1 (S1), Sudan Red G (SRG), Disperse Orange 25 (DO25), Disperse Orange 61 (DO61), Disperse Red 1 (DR1), Disperse Red 13 (DR13) and Disperse Yellow 3 (DY3).

| **Azo dye** | **Organic functional groups (nested) – QSAR Toolbox** | **Log 𝖪_ow_** | | | **Water solubility (mgL^-1^)** | | |
| --- | --- | --- | --- | --- | --- | --- | --- |
|  |  | **Experi-**  **mental** | **KOWWIN v1.68** | **ACD/Labs Percepta logP** | **Experi-**  **mental** | **EPI Suite WaterNT** | **EPI Suite WSKOW** |
| S1 | Azo;  Fused carbocyclic aromatic;  Aryl;  Phenol;  Naphthalene | - | 5.51 | 4.46 | 0.018 (ECHA CHEM) | 4.39 | 0.67 |
| S1 tautomer (stable form) | Aryl;  Hydrazone;  Quinoid compounds | - | 3.312 | 3.81 | - | 4.0 | 36 |
| SRG | Azo;  Ether;  Fused carbocyclic aromatic;  Aryl;  Phenol;  Naphthalene;  Alkoxy | 7.5 (ECHA CHEM) | 5.59 | 4.69 | 0 (ECHA CHEM)  0.00033 (QSAR TB) | 2.3 | 0.38  (0.009 using exp log K_ow_) |
| SRG tautomer (stable form) | Ether;  Aryl;  Hydrazone;  Quinoid compounds;  Alkoxy | - | 3.39 | - | - | 2.1 | 20 |
| DO25 | Azo;  Nitrile;  Aromatic amine;  Nitrobenzene | 4.38 (ECHA CHEM) | 4.69 | 4.46 | <0.02 (ECHA CHEM)    40000 (QSAR TB) | 4.02 | 0.17    (0.33 using exp log K_ow_) |
| DO61 | Azo;  Aryl halide;  Nitrile;  Aromatic amine;  Nitrobenzene | 2.132  (ECHA CHEM) | 6.47 | 5.48 | <20 | 0.17 | 0.00056    (2.8 using exp log K_ow_) |
| DR1 | Alcohol;  Azo;  Aromatic amine;  Nitrobenzene | 4.3 (QSAR TB) | 4.2 | 4.1 | 0.16 (QSAR TB) | 58 | 0.79 |
| DR13 | Alcohol;  Azo;  Aryl halide;  Aromatic amine;  Nitrobenzene | - | 4.85 | 4.68 | 0.012 (QSAR TB) | 13 | 0.16 |
| DY3 | Azo;  Aryl;  Phenol;  Organic amide and thioamide;  Alkyl (hetero)arenes;  Alkyl-, alkenyl- and alkynyl (hetero)arenes | - | 3.98 | 3.27 | 0.00033    1.18 (QSAR TB) | 257 | 10.25 |
| DY3 tautomer | Allyl;  Aryl;  Hydrazone;  Quinoid compounds;  Organic amide and thioamide | - | 1.97 | 1.41 | - | 53 | 386 |


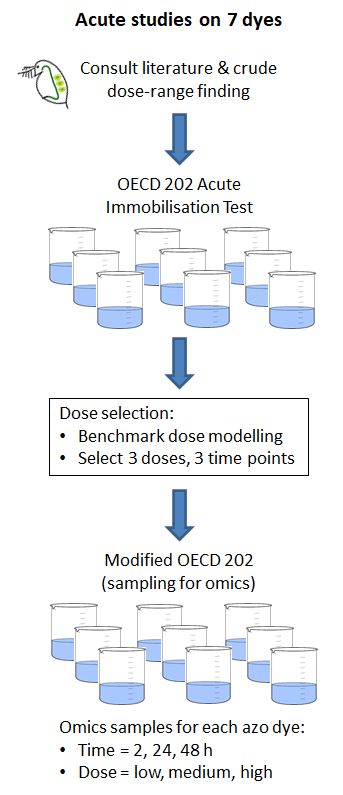


**Fig. S2** Overall workflow for the acute *Daphnia magna* toxicity studies to generate samples for the multi-omics measurements

**Section S1.1. *Daphnia magna* acute exposure studies**

All *D. magna* were maintained under constant environmental conditions (20±1 °C; 16:8-h light:dark photoperiod) in water drawn from a borehole at the University of Birmingham. Cultures (20 *Daphnia* L^-1^) were fed daily with a suspension of *Chlorella vulgaris* corresponding to 0.08 mgC *Daphnia*^-1^. Individuals (<24-h old neonates) used for exposures were obtained from cultures after daily filtration, using the third brood or later. Acute (48 h) toxicity of the azo dyes was determined in accordance with OECD guidelines (OECD, 2004) after consulting published aquatic toxicity data (Table S4) to establish crude dose ranges. Five *D. magna* (<24-h old) in borehole water (200 mL) were exposed in triplicate for 48 h to multiple (≥5) nominal doses of each azo dye (Table S5). Dimethyl sulfoxide (DMSO; Fisher Chemical, UK; >99% purity) was used as carrier solvent for all dyes and untreated controls (final concentration 0.1%). *Daphnia* were not fed during the exposures and observations of immobilisation were recorded after 24 h and 48 h. Benchmark dose (BMD) modelling was conducted on the 48-h immobilisation data using PROASTweb application (version 65.5; <https://proastweb.rivm.nl/>), with a benchmark response (critical effect size) of 0.1. Best-fit models were selected using the lowest Akaike Information Criterion, and the lower and upper estimates of BMD (BMDL and BMDU, respectively) calculated using model-averaging (n = 200).

The multi-omics experimental design included three sampling time points (2-h, 24-h and 48-h) and three dose groups plus untreated controls (0.1% DMSO). Exposures were conducted in six exposure batches (A-F; Table S6) in which all time points and dose groups for a given dye were initiated at the same time with a unique matched control group (except SRG and DR1 shared a control group). Doses were phenotypically anchored to ca. 10% *Daphnia* immobilisation (‘high’ dose), from the BMD modelling; the ‘medium’ and ‘low’ doses were sequentially half-log_10_ (i.e., 3-fold) lower. As DO25 did not induce immobilisation, its high dose was defined by the highest dose used across the other six dyes (Table S6). Therefore, exposures were conducted at equi-effective doses for each of the azo dyes investigated except for DO25. Ten to twelve *D. magna* (<24-h old) in borehole water (200 mL) were exposed in sextuplicate (n=6) vessels per dose and time point. At each sampling time, *D. magna* from the same vessel were collected and divided for metabolomics and transcriptomics (each comprising 5–6 animals per biological sample, n = 6 replicate samples were collected). *Daphnia* were flash-frozen in liquid nitrogen and stored at −80°C until extraction. Only n=4 biological samples were subsequently processed for transcriptomics. Additional untreated animals were collected and used for metabolomics intrastudy quality control (QC) samples. Two 48-h dose groups were discarded for S1 (high and medium doses) and one for DO61 (high dose) due to high *Daphnia* immobilisation during the exposure period (Table S6).

**Table S4.** Existing aquatic toxicity data for the seven azo dyes along with information on their REACH registration status.

| **Azo dye** | **Aquatic toxicity data** | **REACH registered?** | **Notes on registration** |
| --- | --- | --- | --- |
| S1 | *D. magna* 48-h EC50 > 100 mg/L (ECHA dossier)  *D. magna* 21-d LOEC = 60 µg/L, NOEC = 40 µg/L (University of Birmingham experimental data) | Yes | REACH (0-10 tonnes pa)  Notified classification: Aquatic Chronic 4 for chronic toxicity according to the CLP Regulation (EC 1272/2008) |
| SRG | Larval fathead minnows LC50 = 16.7 μg/L (Parrott et al, 2016) | Yes | REACH (0-10 tonnes pa). Justification for waiving the test information in the REACH registration dossier: In accordance with Annex VIII (REACH), testing for aquatic toxicity endpoints is considered scientifically unjustified since there are mitigating factors indicating that aquatic toxicity is unlikely to occur as the substance is highly insoluble (solubility: 0.00033 mg/L at 25° C) in water |
| DO25 | *D. magna* LC50 = 110 mg/L (Brown 1992); zebrafish IC50 = 268 mg/L; *Scenedesmus subspicatus* EC50 = 54 mg/L; Bacteria EC50 > 100 mg/L  *D. magna* 48-h EC50 > 100 mg/L (ECHA dossier) | Yes | REACH (10-100 tonnes pa)  Not classified |
| DO61 | *D. magna* 48-h EC50 = 18.6 mg/L (ECHA dossier)  *D. magna* 21-d LOEC = 1300 µg/L, NOEC = 400 µg/L (University of Birmingham experimental data) | Yes | REACH (10-100 tonnes pa)  Notified classification: Aquatic Chronic Toxicity 3 for chronic toxicity according to the CLP Regulation (EC 1272/2008) |
| DR1 | *D. similis* EC50 = 0.12 mg/L (de Luna 2014); *D. magna* LC50 = 0.58 mg/L, *D. similis* NOEC 14 days = 0.003 mg/L (Vacchi et al 2016); *D. similis* EC50 = 0.13 mg/L (Vacchi et al 2016); *D. similis* EC50 = 0.127 mg/L, NOEC = 0.01 mg/L (Ferraz et al 2011) | No | Not classified for environment |
| DR13 | *D. similis* EC50 = 0.18 mg/L (de Luna 2014);  *D. similis* EC50 = 0.0187 mg/L | No | Not classified for environment |
| DY3 | Aquatic toxicity 96-h PNEC = 0.0023 mg/L from read-across of acute toxicity data for Solvent Yellow 1 (CAS RN 60-09-3) (Environment Canada); Fathead minnow LC50 > 180 mg/L (Little and Lamb 1973) *D. magna* 21-d LOEC = 75 µg/L, NOEC = 40 µg/L (University of Birmingham experimental data) | No | Prohibited in textile materials under EU 2002/371/EC  EU Cosmetics Regulation (1223/2009), Annex II - Prohibited Substances; classification: Carc. 2 |

**Table S5.** Summary of test conditions for the acute dose range-finding study in *Daphnia magna*, and the outputs of the benchmark dose (BMD) modelling of the resulting *D. magna* immobilisation data. These results were used to select exposure doses for the ‘omics exposure study. Lower and upper estimates of BMD corresponding to 10% *Daphnia* immobilisation at 48-h (BMDL and BMDU, respectively) were calculated using PROAST.

| **Azo dye** | **Exposure concentrations (mgL^−1^) in aqueous 0.1% DMSO solution** | **BMDL (mgL^−1^)** | **BMDU (mgL^−1^)** | **Mean of BMDL and BMDU (mgL^−1^) as a dose-anchor for the ‘omics exposures (i.e. ‘equi-effective dose’)** |
| --- | --- | --- | --- | --- |
| S1 | 0, 0.025, 0.050, 0.10, 0.25, 0.50, 1.0 | 0.069 | 0.087 | 0.078 |
| SRG | 0, 0.50, 1.0, 2.5, 5.0, 10.0 | 1.5 | 5.2 | 3.35 |
| DO25 | 0, 0.26, 0.52, 1.04, 2.08, 4.15, 8.3 | - | - | - |
| DO61 | 0, 0.0005, 0.001, 0.005, 0.01, 0.025, 0.05, 0.10, 0.25, 0.50 | 0.0018 | 0.0066 | 0.0042 |
| DR1 | 0, 0.025, 0.05, 0.10, 0.25, 0.50, 1.0 | 0.048 | 0.15 | 0.099 |
| DR13 | 0, 0.01, 0.025, 0.05, 0.10, 0.25, 0.50 | 0.013 | 0.024 | 0.018 |
| DY3 | 0, 0.025, 0.05, 0.10, 0.25, 0.50, 1.0 | 0.86 | 0.97 | 0.91 |

**Table S6.** Summary of test conditions and experimental observations for the *Daphnia magna* acute multi-omics study of seven azo dyes.

| **Azo dye** | **Exposure concentrations (mgL^−1^)** | **Exposure batch design** | **No. of *Daphnia* samples collected for metabolomics + transcriptomics analyses** |
| --- | --- | --- | --- |
| S1 | 0.0087, 0.026, 0.078 | Batch E | 59 + 40 (due to mortality at 48 h, samples discarded) |
| SRG | 0.30, 1.0, 3.35 | Batch B | 72 + 48 (controls shared with DR1) |
| DO25 | 0.30, 1.0, 3.0 | Batch A | 72 + 48 |
| DO61 | 0.0004, 0.0013, 0.004 | Batch C | 69 + 44 (due to mortality at 48 h, samples discarded) |
| DR1 | 0.01, 0.03, 0.10 | Batch B | 72 + 48 (controls shared with SRG) |
| DR13 | 0.002, 0.006, 0.018 | Batch F | 72 + 48 |
| DY3 | 0.10, 0.30, 0.91 | Batch D | 72 + 48 |

**Section S1.2. *Daphnia magna* chronic exposure studies**

*D. magna* chronic (21-d) toxicities of DY3 (target) and S1 (as it was identified as the most suitable source substance, yet lacked reliable chronic toxicity data) was determined in accordance with OECD guidelines (OECD, 2012). Individual *D. magna* (<24-h old) in borehole water (50 mL) were exposed for 21 days to multiple doses of each dye (n = 10 per dose), using nominal doses of 0, 10, 20, 40, 75, 150, 300 and 500 µgL^−1^ for DY3, and 0, 2, 4, 8, 15, 25, 40 and 60 µgL^−1^ for S1. Animals were fed daily with *C. vulgaris* (0.08 mgC *Daphnia*^-1^), with renewal of exposure water every 48 h. The reproductive output of individual *D. magna* was recorded throughout the study, providing a measure of reproductive output. Analysis of variance (ANOVA) followed by a Dunnett’s test was applied to derive a lowest observed effect concentration (LOEC) and no observed effect concentration (NOEC) per dye.

**Section S1.3. Polar and apolar metabolomics: sample extraction, data acquisition, processing and feature annotations**

*Daphnia* samples were collected as described in section 2.3 (main paper), and metabolite extractions were conducted in batches of 12–14 samples, with samples from the six exposure batches randomised across 41 extraction batches. Process blanks containing no biological material were prepared to identify background contaminants from the extraction process. A pooled intrastudy QC sample was prepared by extracting multiple additional *Daphnia* samples, and then splitting this sample into multiple aliquots.

Analytical grade (>98% purity) and LC–MS grade solvents (Honeywell, Merck, UK) were used for all extractions. Solvents were precooled on wet ice. Metabolites were extracted using bead-based homogenisation and biphasic extractions with a final ratio of methanol:chloroform:water of 2:2:1.8 (Taylor et al., 2009). Briefly, frozen *Daphnia* samples were homogenised in methanol (32 µLmg^−1^) and water (10.6 µLmg^−1^) using a Precellys-24 homogeniser (Stretton Scientific; 2 x 10 s bursts at 6400 rpm with a 5 s gap between cycles). Homogenates were transferred to 1.8 mL glass vials, chloroform (16 µLmg^−1^) was added, followed by vortex-mixing for 30 s and incubating on ice for 10 min. Next, further chloroform (16 µLmg^−1^) and water (18.2 µLmg^−1^) were added, vortex-mixed (30 s), and left on wet ice for 10 min to induce phase separation. After centrifugation (4000 rpm, 10 mins at 18°C), polar aliquots (2 x 200 µL) were transferred to Eppendorf tubes and dried overnight in an SPD11V SpeedVac sample concentrator (Thermo Fisher Scientific), and apolar aliquots (2 x 100 µL) transferred to 1.8 mL glass vials and dried under nitrogen for ca. 10 min. All extracts were stored at -80°C.

Dried polar extracts were resuspended in 25 µL 4:1 methanol:water (v/v, Honeywell, Merck) containing 0.1% formic acid (v/v, Merck), and apolar extracts resuspended in 40 µL 2:1 7.5 mM methanolic ammonium acetate:chloroform (v/v, Merck). Extracts were vortexed (30 s), centrifuged (4000 rpm; 15 min; 4°C), and plated into 384-well Eppendorf plates (15 µL for polar and 24 µL for apolar extracts). Due to the high number of samples, resuspended extracts were distributed across three 384-well plates with samples from up to two exposure batches per plate. Biological study samples were randomised within each plate and aliquots of the intrastudy QC were distributed every 8th sample. Nanoelectrospray (nESI) direct infusion mass spectrometry (DIMS) analyses were conducted using an Orbitrap Elite mass spectrometer (Thermo Scientific) in positive ion mode, interfaced with a chip-based nESI platform (Triversa NanoMate, Advion) controlled by ChipSoft software (v8.1.0, Advion). Data acquisition parameters were as previously reported (Southam et al., 2017) with slight modifications for low biomass samples, and data were acquired using Xcalibur software (v2.0, Thermo Scientific).

Analytical procedures adhered to the MEtabolomics standaRds Initiative in Toxicology (MERIT) best practice guidelines (Viant et al., 2019), acquiring data for three types of QC sample: a system suitability QC sample to ensure the mass spectrometer was operating within specification; an intrastudy QC sample to measure the analytical reproducibility and correct for any intra- and inter-batch variation (Kirwan et al., 2014; Southam et al., 2017); and a process blank QC to measure the background signals.

DIMS mass spectra were processed using DIMSPy, implemented in Galaxy (Southam *et al*., 2017; <https://github.com/computational-metabolomics/dimspy-galaxy>). Three stages of feature (or peak) picking and noise filtering were applied as previously reported (Kirwan et al., 2014; Southam et al., 2017), with minor modifications. First, features in the process blank were excluded unless they were >10-fold more intense in the biological samples. This ‘blank-filtered’ feature matrix was copied, and provided the input data for the untargeted ADME/toxicokinetic workflow (described in section 2.5 of the main paper and Section S1.4). Next, the sample filter was set to retain features present in >80% of all biological samples, which removed any azo-dye related features from this endogenous feature matrix. Thirdly, samples with >50% missing values were excluded from further analysis. Next, the data were probabilistic quotient normalised (PQN) on a dye-by-dye basis, due to variation in feature counts across dyes. Missing values were imputed using the k-nearest neighbour approach (k = 5), producing a data matrix for univariate statistical analysis. This matrix was generalised log transformed prior to any multivariate analyses. This workflow resulted in seven endogenous data matrices (one per dye), per DIMS assay. All statistical analyses are described in section 2.7 of the main paper.

Endogenous features were putatively annotated using the Birmingham mEtabolite Annotation for Mass Spectrometry (BEAMS) pipeline (<https://more.bham.ac.uk/beams/>), with an initial mass error tolerance of ±5 ppm. This threshold was determined by examining the system suitability QC data. Subsequently, following inspection of mass error distributions, the error tolerance was reduced to 2 ppm for the apolar DIMS dataset (+0.5 to -1.5 ppm).

**Section S1.4. Untargeted xenobiotic analysis**

Blank-filtered feature matrices from the processing of the DIMS polar and apolar metabolomics datasets served as the input for this workflow, generated using DIMSPy. Subsequent processing was conducted in the R environment (version R-4.0.3), initially applying a three-stage filter to retain only those features arising from the azo dyes and their potential biotransformation products (BTPs):

- Only features present in >80% of all treated high dose biological samples, for each dye, were retained;
- Only features present in <50% of all untreated control samples were retained;
- Only features demonstrating a >10 median fold change in intensity between high dose and untreated control samples were retained.

Next, the data for each individual mass spectrum were normalised to account for relatively subtle differences in the overall mass spectral intensities; this was achieved by applying the scaling factor derived from the PQN normalisation of the endogenous features in the same biological sample.

The resulting dye-related feature intensity matrix was putatively annotated by matching to a list of *in silico* predicted phase I and II BTPs (Systematic Generation of potential Metabolites, SyGMa; score ≥ 0.01% (Ridder & Wagener, 2008)) for each azo dye, using a mass error tolerance of ±10 ppm. Next, the normalised intensities (corresponding to relative concentrations) of the putatively annotated dye-related features were manually inspected for evidence of increasing intensities as a function of increasing nominal exposure dose, the observation of which increased confidence in their putative annotations.

**Section S1.5. Transcriptomics: RNA extraction, data acquisition, processing and gene annotations**

*Daphnia* samples were collected as described in section 2.3 (main paper). Total RNA was extracted from frozen tissue samples using the Agencourt RNAdvance Tissue Kit (Beckman Coulter). Briefly, samples were homogenised in lysis buffer (400 µL lysis buffer + 20 µL proteinase K) using a Geno/Grinder 2020 (Spex), incubated for 25 min (37 °C), transferred to a 96-well plate, sealed and stored at -80 °C. After all samples had been prepared up to this step, the well plates were processed according to the manufacturer’s protocol using a Biomek FXp liquid handling robot (Beckman Coulter). The RNA concentration within each sample was measured by a NanoDrop 8000 spectrophotometer (Thermo Fisher ND-8000-GL). Aliquots of a cross-section of samples were diluted to ~5 ngµL^-1^ and RNA Integrity Number (RIN) was checked using an Agilent Tapestation 2200 (Agilent G2964AA) with High Sensitivity RNA screentape (Agilent 5067- 5579).

For TempO-Seq sequencing, libraries for the targeted gene set are constructed as briefly described in the TempO-Seq^®^ Assay User Guide (BioSpyder Technologies, 2020). PCR-amplified and barcoded samples are pooled into a single library for sequencing using a standard Illumina sequencing platform. Sequencing reads are demultiplexed using the standard sequencing instrument software for each sample using the barcodes and the data are analysed using the Tempo-SeqR software package in R to provide standard FASTQ files for each sample (BioSpyder Technologies, Inc., Carlsbad, CA. United States).

The transcriptomics data consist of the levels at which 1991 targeted protein-coding genes are transcribed in *Daphnia*’s response to the experimental treatments. The gene expression data were obtained by TempO-Seq^®^ on a newly designed *D. magna* BioSpyder platform (<https://biospyder.com/technology/>). Of these 1991 genes, 1885 have homologs to genes found in the human genome representing 1257 unique gene families. By contrast, only 106 genes are unique to the evolutionary lineage leading to *Daphnia*. The methods used to assign genes to homologous gene families that are shared between *Daphnia* and humans are described in (Colbourne et al., 2022). The annotation of the BioSpyder *Daphnia* gene set is given in Online-Resource 4, Table S7. This set was chosen to mirror the S1500+ human gene set that was previously designed for the BioSpyder platform by the Tox21 Working Group of the US National Toxicology Programme (Mav et al., 2018).

In brief, the custom designed probe set consists of *Daphnia* genes with homology to human genes listed within the OrthoDB Database v10 (Kriventseva et al., 2019) to ensure coverage of the curated gene sets and of the canonical pathways. Of the listed 1877 human genes, 1349 (72%) have at least one homolog in *D. magna*. Next, for each of those 788 gene families that contained more than a single *D. magna* homolog (i.e. by gene duplications), their genes were clustered using Euclidean distance and Ward’s linkage method based on the similarity of their gene expression patterns across 99 RNA-Seq measurements, including those from a pilot study of *D. magna* gene expression during the early (4-h) and late (48-h) response to exposure to a single azo dye, DR1 (data not shown). By this method, the similarity measurements are the Euclidean distances among genes implemented using JMP® (SAS Institute Inc.). A single *D. magna* gene was chosen from each multi-gene cluster that differed in expression pattern from another cluster of the gene family by a distance greater than 5. This step raised the number of *D. magna* genes, mapped to the S1500+ human gene set, to 1604. The remaining 387 genes included on the platform were chosen based on their expression patterns to specific toxicological conditions of interest.

TempO-Seq transcriptomics data consists of a raw count matrix for probes (2378 probes covering possible gene isoforms) against individual samples. Counts were first summarised to (unique) *D. magna* genes covered on the platform (1988 genes) and a total of 6 genes were removed due to high nonspecific background counts (false positive signal) or cross-gene hybridisation, resulting in a summarised count matrix covering 1982 genes. Next, reads with low counts (< 10 across all dye samples) were removed from the dataset (17 genes). Principal component analysis (PCA) was initially performed on r-logged counts to identify potential outliers that were removed from subsequent analyses. Count normalisation and differential expression (DE) analysis were conducted in R (version R-4.0.3) using the *DESeq2* package (version 1.30.0; (Love et al., 2014)). Final analyses were performed on treated and control samples for each dye separately, treating each dose/time combination as a separate sample type.

**Table S7.** Annotation of the custom BioSpyder *Daphnia magna* gene set.

See file “Table S7.xlsx” in Online Resource 4.


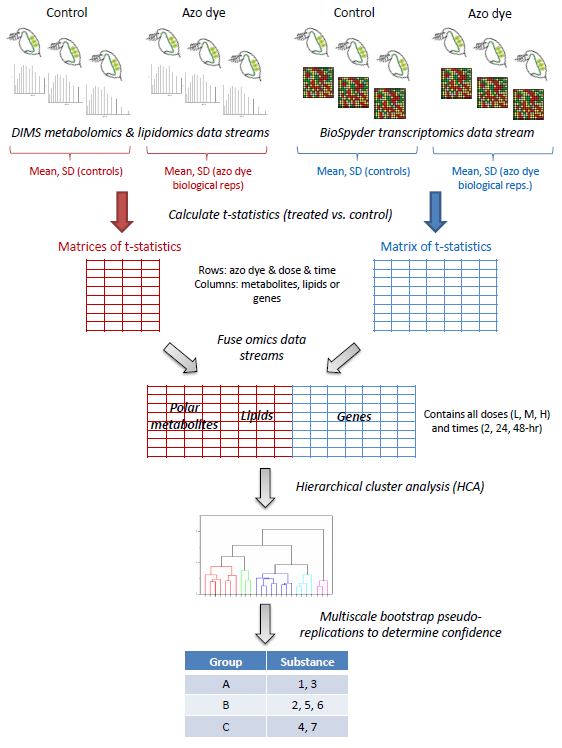


**Fig. S3** Overview of the processing and statistical analysis of the multi-omics data within the bioactivity profile-based grouping workflow

## 2. Results & Discussion

**Section S2.1. Assimilate and compare physico-chemical properties of the seven dyes**

The physico-chemical properties of the seven azo dyes are summarised in Table S3. All seven dyes have rather low water solubility. Water solubility predicted with WSKOWappears to be closer to the experimentally-determined water solubility. WSKOW predicts water solubilities between 0.1 and 1 mgL^-1^ for both Sudan dyes (S1 and SRG), for both Disperse Red dyes (DR1 and DR13), and for DO25. Water solubility is very low for DO61, and more than 10 mgL^-1^ for DY3. If it is the case that these azo dyes have no specific MoA (i.e., act as narcotics), then their toxicities could be related to log K_ow_. In which case the Sudan dyes (S1 and SRG) should exhibit higher toxicity compared to DY3, DR1, DR13 and DO25. For DO61 there is conflicting information between experimental data and predicted log Kow, the latter suggesting the highest log K_ow_ and hence the highest narcotic toxicity of all of the dyes. In addition, the Sudan dyes and DY3 give an alert for tautomerisation in the QSAR Toolbox. The tautomers could have different properties, however it is unclear if in practice these would be observed.


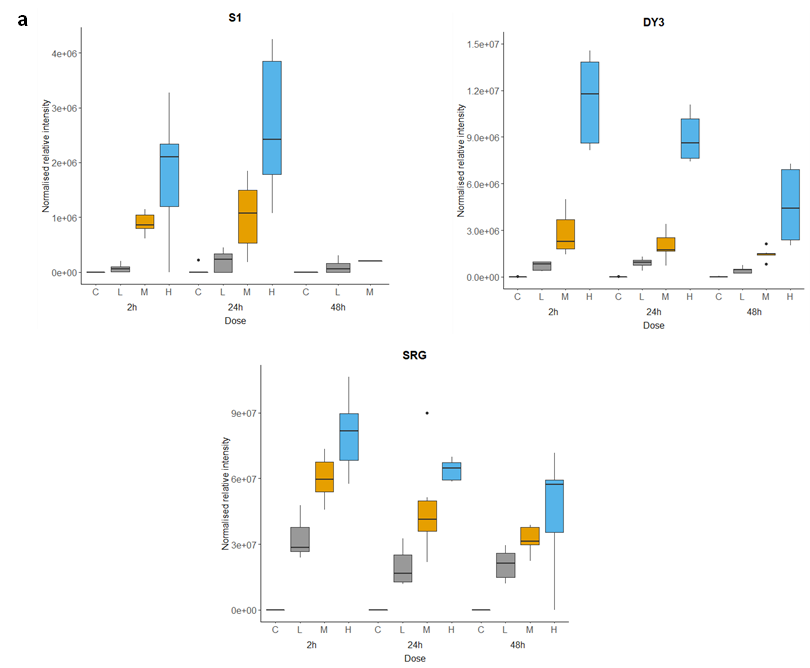


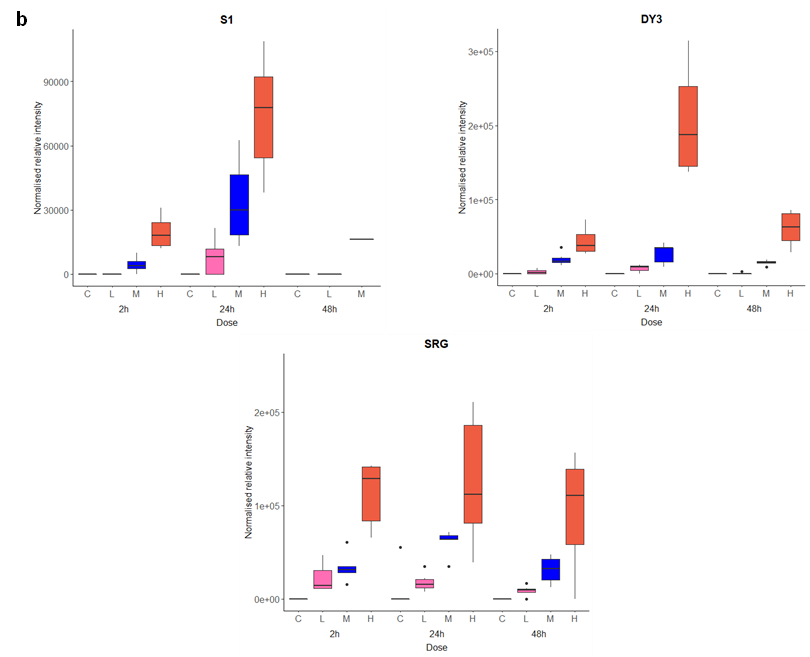


**Fig. S4** Normalised intensities of three azo dyes (S1, DY3 and SRG) measured in (a) apolar extracts, and (b) polar extracts of *Daphnia magna*, across the three nominal exposure doses (C – control, L – low, M – medium, and H – high) and three sampling time points (2-, 24- and 48-h). Data was not measured for the 48-h high-dose time point for S1, due to high *D. magna* immobilisation

**Table S8.** Measurements of seven azo dyes and their metabolic biotransformation products in the apolar extracts of *Daphnia magna* (positive ion mode DIMS dataset), including putative annotations, *m/z* data and the outputs from the filtering criteria used in the untargeted xenobiotic workflow. Correlation between analyte relative intensity and the nominal exposure dose (low, medium or high) was flagged as ‘yes’ if it occurred in at least one time point (of the 2-, 24- and 48-h exposures).

| **Azo dye** | **Parent or biotransformation product (BTP)** | **Theoretical *m/z* of [M+H]^+^ ion** | **Measured *m/z* of [M+H]^+^ ion** | **Absolute mass error (ppm)** | **% presence in high dose samples** | **% presence in control samples^[[1]](#footnote-1)^** | **Median fold-change intensity (high vs. control)** | **Intensity correlated with exposure dose?** |
| --- | --- | --- | --- | --- | --- | --- | --- | --- |
| DY3 | Parent | 270.12370 | 270.12398 | 1.0 | 100% | 28% | 199 | Yes |
|  | BTP1 - N-deacetylation | 228.11314 | 228.11378 | 2.8 | 100% | 0% | ∞ | Yes |
|  | BTP2 - Methylation | 284.13935 | 284.13979 | 1.5 | 94% | 0% | ∞ | No |
|  | BTP3 - Aromatic hydroxylation | 286.11862 | 286.11906 | 1.5 | 100% | 0% | ∞ | No |
|  | BTP4 - N-deacetylation + sulfation | 308.06995 | 308.07129 | 4.3 | 94% | 0% | ∞ | No |
| S1 | Parent | 249.10223 | 249.10364 | 5.7 | 82% | 6% | 10.7 | Yes |
|  | BTP1 - Aromatic hydroxylation | 265.09715 | 265.09864 | 5.6 | 82% | 0% | ∞ | Yes |
| SRG | Parent | 279.11280 | 279.11286 | 0.2 | 89% | 0% | ∞ | Yes |
|  | BTP1 - Methylation | 293.12845 | 293.12917 | 2.4 | 89% | 0% | ∞ | Yes |
| SRG | BTP2 - Aromatic hydroxylation | 295.10772 | 295.10858 | 2.9 | 89% | 0% | ∞ | Yes |
| DR1 | Parent | 315.14517 | 315.14595 | 2.5 | 100% | 0% | ∞ | Yes |
| DR13 | Parent | 349.10619 | 349.10807 | 5.4 | 100% | 0% | ∞ | Yes |
| DO25 | Parent (tentative, low fold change) | 324.14550 | 324.14551 | 0.03 | 100% | 17% | 2.3 | Yes |
|  | BTP1 - Nitrile to amide (tentative, low fold change) | 342.15607 | 342.15681 | 2.2 | 100% | 17% | 3.3 | Yes |
| DO61 | Parent | 481.96457 | 481.96487 | 0.6 | 100% | 0% | ∞ | Yes |

**Table S9.** Measurements of five azo dyes and their metabolic biotransformation products in the polar extracts of *Daphnia magna* (positive ion mode DIMS metabolomics dataset), including putative annotations, *m/z* data and the outputs from the filtering criteria used in the untargeted xenobiotic workflow. Correlation between analyte relative intensity and the nominal exposure dose (low, medium or high) was flagged as ‘yes’ if it occurred in at least one time point (of the 2-, 24- and 48-h exposures).

| **Azo dye** | **Biotransformation product (BTP)** | **Theoretical *m/z* of [M+H]^+^ ion** | **Measured *m/z* of [M+H]^+^ ion** | **Absolute mass error (ppm)** | **% presence in high dose samples** | **% presence in control samples^[[2]](#footnote-2)^** | **Median fold-change intensity (high vs. control)** | **Intensity correlated with exposure dose?** |
| --- | --- | --- | --- | --- | --- | --- | --- | --- |
| DY3 | Parent | 270.12370 | 270.12485 | 4.3 | 100% | 0% | ∞ | Yes |
|  | BTP1 - N-deacetylation + sulfation | 308.06995 | 308.07216 | 7.2 | 100% | 0% | ∞ | Yes |
|  | BTP2 - N-deacetylation | 228.11313 | 228.11466 | 6.7 | 100% | 11% | 19.0 | Yes |
| S1 | Parent | 249.10223 | 249.10273 | 2.0 | 100% | 0% | ∞ | Yes |
| SRG | Parent (tentative, low fold change) | 279.11280 | 279.11471 | 6.8 | 94% | 6% | 2.3 | Yes |
|  | BTP1 - Aromatic hydroxylation | 295.10771 | 295.10984 | 7.2 | 94% | 0% | ∞ | Yes |
| DR1 | Parent | 315.14516 | 315.14770 | 8.1 | 94% | 0% | ∞ | No |
| DR13 | - | - | - | - | - | - | - | - |
| DO25 | Parent | 324.14550 | 324.14758 | 6.4 | 94% | 44% | 48.3 | No |
| DO61 | - | - | - | - | - | - | - | - |


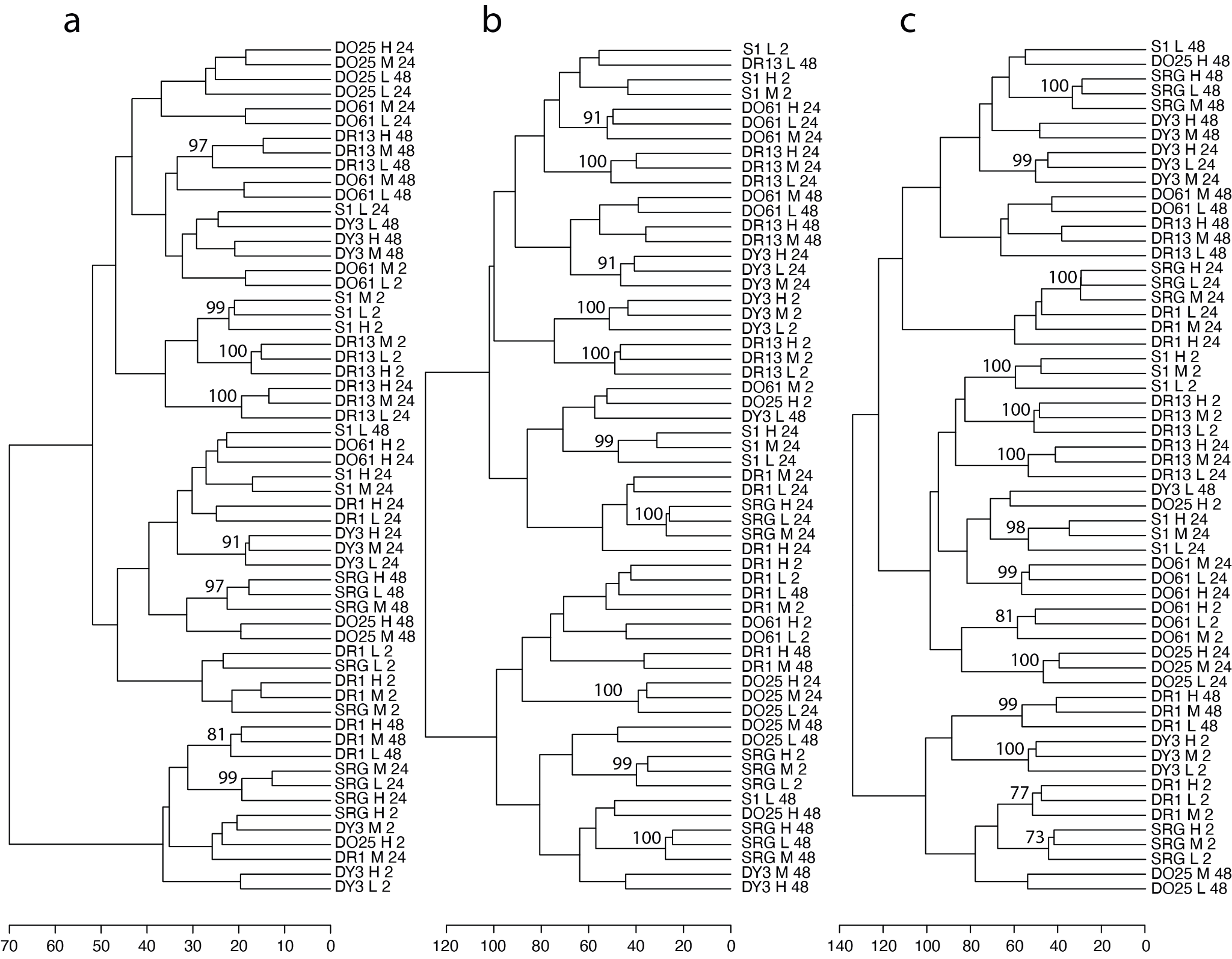


**Fig. S5** Dendrograms produced by the HCA grouping workflow using t-statistics derived from polar and apolar metabolomics data consisting of 428 features (a), transcriptomics data consisting of 1889 features (b) and all three ‘omics datasets combined (c), from samples of *Daphnia magna* neonates collected after 2-h, 24-h and 48-h exposures to low, medium and high doses of seven azo dyes (DY3, S1, SRG, DR1, DR13, DO25, DO61), corresponding to 58 treatment groups. X-axis values indicate the distance measurements (the sum of branch lengths) among any pair of doses, exposure times and substances. The values at the top of the branches indicate % bootstrap replicability confidence (using approximately unbiased (AU) tests of non-selective inference) for nodes that are grouping all three doses of a given dye (at a specific time point), which is an anticipated result given that these three doses span less than one order of magnitude. An accounting of the groupings of all three doses indicates that (1) combined ‘omics data at 2-h grouped all three doses; (2) combined ‘omics data at 24-h grouped all three doses except for DR1, however these all stem from a common node (with SRG); (3) combined ‘omics data at 48-h grouped all three doses only for two dyes (SRG, DR1)

**Table S10.** Mapping of the 33 non-zero structural fragments of ToxPrint chemotypes onto the dendrograms derived from structure-based grouping (Fig. 1) and ‘omics bioactivity profile-based grouping (Fig. 3). This highlights that informative structural data are sparse, as only 33 of the 729 parameters encoded in a ToxPrint chemotype are non-zero. Such a small dataset may not lead to a reliable grouping hypothesis and interpretation, especially if inappropriate confidence metrics are used. Mapping the ToxPrint chemotypes onto the bioactivity profile-based grouping, we detected two structural fragments that may drive the similar molecular responses observed for DY3, S1 and SRG, and two further structural fragments that may drive the similar molecular responses for DO61 and DR13. Therefore, 4/33 (12%) of the chemically informative fragments are potentially biologically informative (4/729 or 0.5% of the ToxPrint chemotype).

| **Grouping hypotheses** | **Structure feature(s) that support the grouping** |
| --- | --- |
| S1 and SRG  (from Fig. 1, based on structure) | Only a single chemical fragment: ring:fused_[6_6]_naphthalene_C1=CC2=C(C=C1)C=CC=C2 (33^rd^ column in Fig. 4) |
| DO25 and DO61  (from Fig. 1, based on structure) | Three chemical fragments: bond:C#N_nitrile_generic (1^st^ column in Fig. 4), bond:C#N_nitrile (2^nd^ column), bond:X[any]_halide (26^th^ column) |
| DR1 and DR13  (from Fig. 1, based on structure) | Four chemical fragments: bond:CN_amine_alkyl_ethanolamine (7^th^ column in Fig. 4), bond:COH_alcohol_aliphatic_generic (14^th^ column), bond:COH_alcohol_pri-alkyl (18^th^ column), group:ligand_path_4_bidentate_aminoethanol (30^th^ column) |
| DY3, S1 and SRG  (from Fig. 3, based on ‘omics bioactivity) | Two chemical fragments: COH alcohol aromatic bond (15^th^ column in Fig. 4), COH alcohol aromatic phenol bond (16^th^ column) |
| DO61 and DR13  (from Fig. 3, based on ‘omics bioactivity) | Two chemical fragments: bond:CX_halide_aromatic-X_generic (20^th^ column in Fig. 4), bond:X[any]_halide (24^th^ column) |

## References

BioSpyder Technologies. (2020). *TempO-Seq ® Assay User Guide (January 15, 2020)*. https://static1.squarespace.com/static/5dcd844d0244664b3eaed21c/t/5ebefdbecb43e454798b23ea/1589575104566/TempO-Seq+User+Guide+%28200115%29.pdf).

Colbourne, J. K., Shaw, J. R., Sostare, E., Rivetti, C., Derelle, R., Barnett, R., Campos, B., LaLone, C., Viant, M. R., & Hodges, G. (2022). Toxicity by descent: A comparative approach for chemical hazard assessment. *Environmental Advances*, *9*, 100287. https://doi.org/10.1016/J.ENVADV.2022.100287

Kirwan, J. A., Weber, R. J. M., Broadhurst, D. I., & Viant, M. R. (2014). Direct infusion mass spectrometry metabolomics dataset: a benchmark for data processing and quality control. *Scientific Data*, *1*(1), 140012. https://doi.org/10.1038/sdata.2014.12

Kriventseva, E. V, Kuznetsov, D., Tegenfeldt, F., Manni, M., Dias, R., Simão, F. A., & Zdobnov, E. M. (2019). OrthoDB v10: sampling the diversity of animal, plant, fungal, protist, bacterial and viral genomes for evolutionary and functional annotations of orthologs. *Nucleic Acids Research*, *47*(D1), D807–D811. https://doi.org/10.1093/nar/gky1053

Love, M. I., Huber, W., & Anders, S. (2014). Moderated estimation of fold change and dispersion for RNA-seq data with DESeq2. *Genome Biology*, *15*(12), 550. https://doi.org/10.1186/s13059-014-0550-8

Mav, D., Shah, R. R., Howard, B. E., Auerbach, S. S., Bushel, P. R., Collins, J. B., Gerhold, D. L., Judson, R. S., Karmaus, A. L., Maull, E. A., Mendrick, D. L., Merrick, B. A., Sipes, N. S., Svoboda, D., & Paules, R. S. (2018). A hybrid gene selection approach to create the S1500+ targeted gene sets for use in high-throughput transcriptomics. *PLOS ONE*, *13*(2), e0191105-. https://doi.org/10.1371/journal.pone.0191105

OECD. (2004). *Test No. 202: Daphnia sp. Acute Immobilisation Test, OECD Guidelines for the Testing of Chemicals, Section 2*. OECD Publishing, Paris. https://doi.org/10.1787/9789264069947-en

OECD. (2012). *Test No. 211: Daphnia magna Reproduction Test, OECD Guidelines for the Testing of Chemicals, Section 2*. OECD Publishing, Paris. https://doi.org/10.1787/9789264185203-en

Ridder, L., & Wagener, M. (2008). SyGMa: Combining Expert Knowledge and Empirical Scoring in the Prediction of Metabolites. *ChemMedChem*, *3*(5), 821–832. https://doi.org/https://doi.org/10.1002/cmdc.200700312

Southam, A. D., Weber, R. J. M., Engel, J., Jones, M. R., & Viant, M. R. (2017). A complete workflow for high-resolution spectral-stitching nanoelectrospray direct-infusion mass-spectrometry-based metabolomics and lipidomics. *Nature Protocols*, *12*(2), 310–328. https://doi.org/10.1038/nprot.2016.156

Taylor, N. S., Weber, R. J. M., Southam, A. D., Payne, T. G., Hrydziuszko, O., Arvanitis, T. N., & Viant, M. R. (2009). A new approach to toxicity testing in Daphnia magna: application of high throughput FT-ICR mass spectrometry metabolomics. *Metabolomics*, *5*(1), 44–58. https://doi.org/10.1007/s11306-008-0133-3

Viant, M. R., Ebbels, T. M. D., Beger, R. D., Ekman, D. R., Epps, D. J. T., Kamp, H., Leonards, P. E. G., Loizou, G. D., MacRae, J. I., van Ravenzwaay, B., Rocca-Serra, P., Salek, R. M., Walk, T., & Weber, R. J. M. (2019). Use cases, best practice and reporting standards for metabolomics in regulatory toxicology. *Nature Communications*, *10*(1), 3041. https://doi.org/10.1038/s41467-019-10900-y

1. Features detected in control samples most likely indicate low intensity isobaric endogenous species, but could also arise from noise within the mass spectra and/or contamination with parent dyes. [↑](#footnote-ref-1)
2. Features detected in control samples most likely indicate low intensity isobaric endogenous species, but could also arise from noise within the mass spectra and/or contamination with parent dyes. [↑](#footnote-ref-2)
